# Enhance nisin yield via improving acid-tolerant capability of *Lactococcus lactis* F44

**DOI:** 10.1038/srep27973

**Published:** 2016-06-16

**Authors:** Jian Zhang, Qinggele Caiyin, Wenjing Feng, Xiuli Zhao, Bin Qiao, Guangrong Zhao, Jianjun Qiao

**Affiliations:** 1 Department of Pharmaceutical Engineering, School of Chemical Engineering and Technology, Tianjin University, Tianjin 300072, China; 2 Key Laboratory of Systems Bioengineering, Ministry of Education Tianjin, 300072, China; 3 SynBio Research Platform, Collaborative Innovation Center of Chemical Science and Engineering, Tianjin 300072, China

## Abstract

Traditionally, nisin was produced industrially by using *Lactococcus lactis* in the neutral fermentation process. However, nisin showed higher activity in the acidic environment. How to balance the pH value for bacterial normal growth and nisin activity might be the key problem. In this study, 17 acid-tolerant genes and 6 lactic acid synthetic genes were introduced in *L. lactis* F44, respectively. Comparing to the 2810 IU/mL nisin yield of the original strain F44, the nisin titer of the engineered strains over-expressing *hdeAB*, *ldh* and *murG*, increased to 3850, 3979 and 4377 IU/mL, respectively. These engineered strains showed more stable intracellular pH value during the fermentation process. Improvement of lactate production could partly provide the extra energy for the expression of acid tolerance genes during growth. Co-overexpression of *hdeAB*, *murG*, and *ldh(Z)* in strain F44 resulted in the nisin titer of 4913 IU/mL. The engineered strain (ABGL) could grow on plates with pH 4.2, comparing to the surviving pH 4.6 of strain F44. The fed-batch fermentation showed nisin titer of the co-expression *L. lactis* strain could reach 5563 IU/mL with lower pH condition and longer cultivation time. This work provides a novel strategy of constructing robust strains for use in industry process.

Nisin, as an antimicrobial peptide with 34 residues, is known for its wide range of antimicrobial activity against gram-positive bacteria and also shows antimicrobial activity against gram-negative bacteria especially when combined with a chelating agent, such as disodium EDTA^1^,^2^. Therefore it is widely applied in the dairy and meat manufacturing industries for its outstanding characteristics of higher antimicrobial efficiency, less poisonous compared to other chemical preservatives^3^. Due to its wide commercial application, many methods and technologies have been adopted to improve nisin yield and further reduce production cost, such as intentional mutagenesis^4^, optimization of fermentation process^5^, traditional random mutation and genome shuffling^6^, etc. In previous studies, some approaches was attempted to increase nisin production by immobilization of *L. lactis* cells and optimization of the nisin pathway for nisin biosynthesis. Expression of chitin-binding domain (ChBD) from *Bacillus circulans* which was fused with PrtP anchor on the surface of *L. lactis* for specific binding to chitin could maintain high cell densities and stable nisin production^7^, the highest nisin production was improved to 10500 IU/mL by the continuous nisin production system CICON-FER^8^. In *Lactococcus lactis* subsp. *lactis* A164, additional copies of nisin self-protection (immunity) genes *nisFEG* and the regulatory genes *nisRK* with high copy number plasmid in the producer strain enhanced the 1.5–1.7-fold higher than the nisin yield of the original strain^9^. Also in another study, provision of additional copies by cloning *nisRK* and/or *nisFEG* genes on plasmids in the *L. lactis* LL27 resulted in producing 45%, 24% and 44% more nisin compared to the original yield 520 IU/mL, respectively^10^. Furthermore, the bioactive recombinant nisin of the nisin deficient strain *L. lactis* MG1363 were overproduced with a yield of 1098 IU/mL when additional copies of an entire nisin biosynthesis pathway were present^11^.

Despite many methods and technologies exploited before, the requirement to construct a manufacturing strain based on fermentation process is unsatisfied. *L. lactis*, as one of main nisin producers, is inhibited by lactic acid produced during the fermentation process. Hence fermentation condition has to be neutralized in order to ensure the cell growth in traditional fermentation process^12^. However, nisin, a significant product, showed a relative higher activity in the environment with the pH 2.0 or lower and degraded as the increase of pH value^6^. To alleviate the degradation rate of nisin during the fermentation process, lowering pH value of the fermentation broth will be an alternative approach. Therefore, it is necessary to construct an acid tolerant strain which can maintain its growth activity in the relative acidic fermentation condition, and also alleviate nisin degradation.

Fortunately, recent advances in genome mining methods have provided a potential access to disclose acid tolerant genes and gene clusters^13^,^14^. A number of acid tolerant mechanisms in many microorganisms have been elucidated, such as in *L. Lactis* and *E. coli*, which make it possible to improve acid tolerance of bacteria by the transformation of acid tolerant genes. The mechanisms of acid tolerance in bacteria can be classified into four categories: (I) Decrease of intracellular protons mainly involved in transmitting obstacle of extracellular H^+^, exportation of intracellular H^+^ and consumption of intracellular H^+^. (Ia) The solidification of cell wall, the change of cell membrane and the protection of trehalose can work by transmitting obstacles of extracellular H^+ ^15,16,17,18,19,20. (Ib) The mechanism of intracellular H^+^ consumption mainly includes decarboxylation, the generation of alkali and the protective effect of glutathione and histidine^21^,^22^,^23^,^24^,^25^,^26^,^27^. (Ic) Proton pumps plays a major role in exportation of intracellular H^+^. The multi-subunit F_1_F_0_-ATPase can generate ATPs which facilitate the extrusion of proton from the cell^28^,^29^. (II) Molecular chaperone family proteins, such as 70 kDa DnaK family and 60 kDa GroE family proteins, can play protective roles under acid stress^30^. In addition, the protection of DNA repair for cells are needed. Studies shown that DNA synthesis and repair increased under acid stress^31^,^32^. (III) Regulatory factors include the global regulatory factors, two-component-regulating system and other regulatory factors. (IIIa) EvgS/EvgA is the main two-component signal transduction systems (TCS) in the *E. coli*, which can endow bacteria the sense ability of acid stress and is involved with the regulation of other genes^33^,^34^. (IIIb) The overexpression of some non-coding sRNAs can improve the ability against low pH environment significantly. A novel sRNA gcyB can increase the survival of *E. coli* by enhancing the expression level of sigma factor RpoS in the stationary phase^35^. Simultaneous overexpression of the non-coding sRNA, such as DsrA, RprA and ArcZ displayed an 8500-fold higher survival of *E. coli* to low pH, which also provided a protective effect on carboxylic acid and oxygen stress^36^. (IIIc) Sigma factors including RpoS belong to the global regulatory factors. Sigma factors ensure RNA polymerase combined to specific region of promoters, and RpoS can protect bacterial strains against various stress conditions such as heat shock, acid or alkali stress. The number of regulated genes by RpoS is more than 500 according to the literature^37^. (IV) Some membrane proteins related with carbon metabolism also could contribute to the acid tolerance of bacterial strains^38^,^39^,^40^,^41^. In response to low pH, the central carbon metabolism would be strengthened^42^. More energy can be obtained from the enhanced carbohydrate metabolism, which can be consumed by the bacterial strains against the acid stress.

This study aims to construct an acid tolerant module in *L. lactis*, hoping to balance the optimal pH of cell growth and nisin activity, so as to obtain higher nisin yield. To reach this goal, some acid tolerant genes were transformed to *L. lactis* F44, and the nisin production was analyzed.

Acid tolerance tests of these overexpression strains demonstrated that the nisin production was positively correlated with the heterologous acid tolerant genes. Higher nisin yield and growth of strains could be maintained simultaneously. To our knowledge, this is the first report to reveal the relationship between nisin production and acid tolerance during fermentation process. This work represents a new strategy to introduce process-based-design concept into constructing robust industrial strain.

## Results

### Activity of nisin and the growth of the original strain in acidic condition

**Figure 1:**
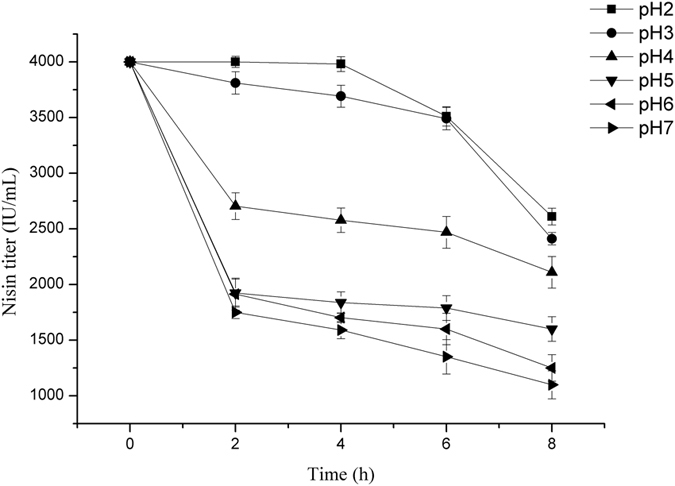
The degradation curves of nisin in the fermentation medium with different pH value. The nisin activity assay was carried out in 250-mL Erlenmeyer flasks containing 100 mL fermentation medium for 8 h at 30 °C.

Nisin, as a kind of small molecular peptide, showed different activity in diverse pH conditions. For analyzing the activity of nisin in fermentation process, 4000 IU/mL nisin standards were added to the fermentation medium at pH 2.0, 3.0, 4.0, 5.0, 6.0 and 7.0, respectively. Nisin titer was measured every 2 hours during the incubation. As it is shown in Fig. 1, the activity of nisin decreased significantly with the increase of pH value of medium. Nisin could remain stable for 4 h at pH 2.0, but when the pH value reached 5.0, nisin titer fell by 50% in 2 h.

Fermentation process was also greatly affected by the initial pH of broth. The fermentation medium was adjusted to pH 2.0, 3.0, 4.0, 5.0, 6.0 and 7.0, respectively, and the starter strain *L. lactis* F44 was incubated for 12 h in above media respectively. Biomass, pH and nisin titer of the fermentation broth were measured every two hours during fermentation process. As shown in Supplementary Fig. S1a, the growth status of the bacteria was declining with the pH values decreasing. When the pH value of broth was under 4.0, the growth of F44 stopped. During fermentation process, the pH value of the fermentation broth reduced because of lactic acid production (see Supplementary Fig. S1b). Nisin accumulation began from 2 h, and reached to its peak of 2810 IU/mL at 8 h, and then declined (see Supplementary Fig. S1c). At the end of the fermentation, the nisin titer reduced by 50% compared with the highest. The optical density of F44 reached maximum at 8 h, meanwhile, the pH value was close to 5.0, which was unfavorable for the growth of F44 strain, thus led to the growth decrease and the nisin degradation.

Nisin can be preserved better below pH 2.0 conditions, and the activity of nisin reduced with the increase of pH. While the strain F44 can hardly grow below pH 5.0. The optimal pH difference between nisin activity and bacteria growth limited the nisin production. Thus, it would be a good method to solve the problem by improving acid tolerance of *L. lactis* strains.

### Introduction of single acid tolerant gene to improve nisin production

**Figure 2:**
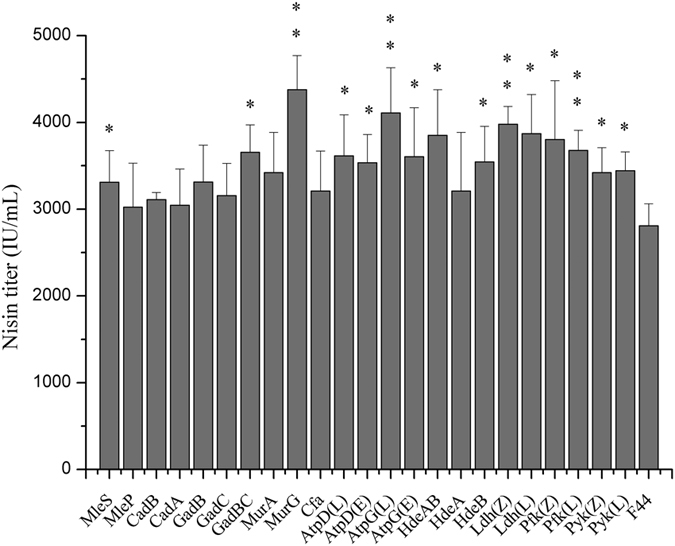
Effect of different acid tolerance genes on nisin titer in 8h (at the peak value). Original strain F44, strains overexpressing acid tolerant genes and strains overexpressing lactic acid synthetic genes were grown in feed medium at 30 °C, and photographed every 2 h. Comparative nisin production in F44 and engineered strains. Error bars, SD from three replicate flasks. *p < 0.05, **p < 0.01, t-test.

To determine the effects of different acid tolerant proteins on nisin production, various acid tolerant genes with different mechanism were introduced respectively into *L. lactis* F44 and the effects of these genes on acid tolerance were analyzed. Firstly, genes with different acid tolerant mechanisms were transformed to *L. lactis* F44 strain individually. It is indicated that the capacity of nisin production increased differently due to the expression of different acid tolerant genes (Fig. 2). Among the engineered strains, the strain harboring *murG* gene, which is responsible for the transfers of the N-acetylglucosamine moieties onto the carrier lipid in cell wall biosynthesis^43^, performed best in nisin production. Nisin accumulation was up to 4377 IU/ml, which is 55.7% higher than that of the original strain. Meanwhile, the growth rate of the engineered strains was slightly lower than that of F44 (see Supplementary Table S3). At the late fermentation period, such as 8 h after starting fermentation, F44 stopped growing almost, while the engineered strains can still maintain good growth activity at least till 10 h.

### Enhancing lactic acid synthesis pathway to improve nisin production

We also cloned two groups of *pyk*, *pfy* and *ldh* genes encoding pyruvate kinase Pyk, phosphofructokinase Pfk and lactate dehydrogenase Ldh, the key enzymes of lactic acid synthesis pathway, from *L. lactis* F44 and *Lactobacillus casei* Zhang, respectively (Fig. 2). Then six genes were introduced to *L. lactis* F44 separately. The results showed that nisin titer of the engineered strains harboring *pyk*, *pfy* and *ldh* all have been improved. Among the engineered strains, the nisin titer of F44 overexpressing *ldh* gene from *L. casei* Zhang was highest. The nisin titer was improved from 2810 IU/mL to 3979 IU/mL, increased by 41.6%. As for the cell growth, the six strains performed similarly with the strains harboring single acid tolerant gene, which showed better robustness than F44 in the stationary phase (see Supplementary Fig. S2a,b).

### Combined expression of several genes to improve nisin production

**Figure 3:**
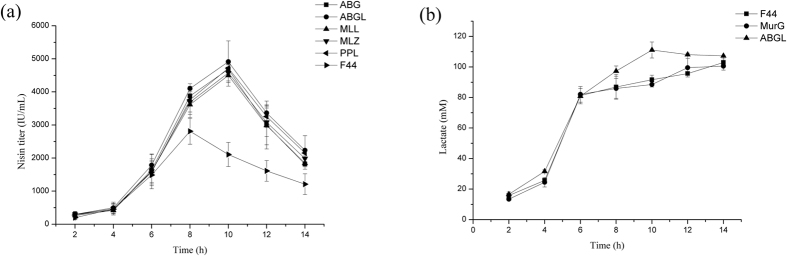
Effect of several acid tolerance genes overexpression on nisin titer (**a**) and lactate production (**b**). Original strain F44, co-overexpression strains were grown in feed medium at 30 °C, and photographed every 2 h. Error bars, SD from three replicate flasks.

**Figure 4:**
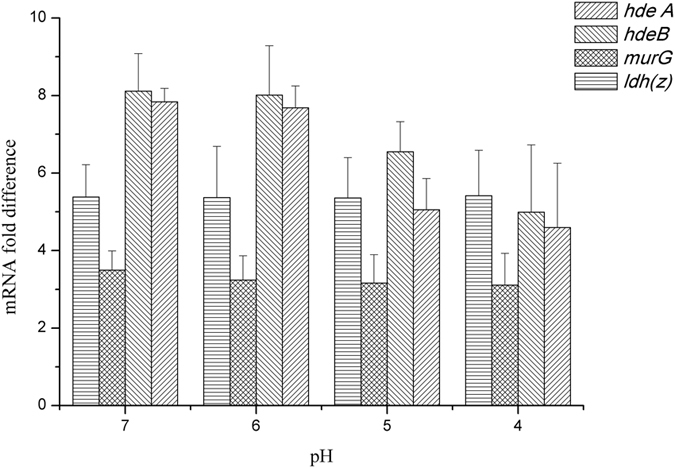
The transcriptional analysis of acid-tolerant genes *hdeA*, *hdeB murG* and *ldh* in wild type F44 and the engineered strain F44 (ABGL) by qRT-PCR. The total RNA extracted from cells which were incubated at pH 7.0, 6.0, 5.0 and 4.0 in seed medium, respectively. The obtained cDNA was subjected to qRT-PCR. The 16S rRNA gene was used as the internal control gene, and *hdeA*, *hdeB murG* and *ldh* in wild type F44 were set as the guide sample.

To further improve the acid tolerance of the strain, the genes which helped to enhance the nisin yield of *L. lactis* F44 through single overexpression and involves in different acid tolerant mechanisms and energy metabolism were selected to be co-overexpressed, and we constructed the following overexpression strain: *L. lactis* F44 (ABG) (combined expression of triple genes *hdeA*, *hdeB* and *murG*), F44 (ABGL) (combined expression of tetrad genes, *hdeA*, *hdeB*, *murG* and *ldh* (Z)), F44 (MLL) (combined expression of *murG* and *ldh* (L)), F44 (MLZ) (combined expression of *murG* and *ldh* (Z)), F44 (PPL) (combined expression of triple genes, *pfk* (Z), *pyk* (Z) and *ldh* (Z)). The nisin titer of all these engineered strains increased during the fermentation process (Fig. 3a). However, at the exponential time, the growth rate of the engineered strains was significantly lower than F44 strain. This might result from the extra-consumption of energy in over-expression of acid tolerant genes, and shortage of energy used in cell growth. When entering the late exponential phase, strain F44 (ABGL) showed remarkable increase in the production of lactate, which could provide bacteria more energy (Fig. 3b). The extra energy supply might be used to maintain the stability of cells. Among the engineered strains, the highest nisin titer was observed for F44 (ABGL), which displayed 4913 IU/mL nisin titer, increased by 74.84% compared with F44 strain. In addition, the transcription levels of *hdeA*, *hdeB*, *murG* and *ldh*(Z) in the F44 (ABGL) were significantly higher than its corresponding genes in the wild type F44 (Fig. 4a,b). The efficiency of the PCR reached 90.13% (see Supplementary Fig. S4), which meant the results of qRT-PCR analysis showed that the P45 promoter of pLEB124 had high efficiency at different pHs and all the heterogenous genes in the engineered strains could be successfully transcribed to the corresponding mRNAs.

The expression levels of the nisin biosynthesis-related genes between wild type F44 and the engineered strain F44 (ABGL) were examined by qRT-PCR. The cells at mid-log phase (8 h) for the highest gene expression was harvested for qRT-PCR. As shown in Supplementary Fig. S6, the expression levels of most genes related to nisin biosynthesis of F44 (ABGL) were slightly higher than that of the original strain F44. And the change of expression levels between the two strains was less than 2 times, which demonstrated no significant differences were found. Moreover, the expression levels of other nisin related genes *nisB* and *nisR* of F44 (ABGL) were lower than that of F44.

**Figure 5:**
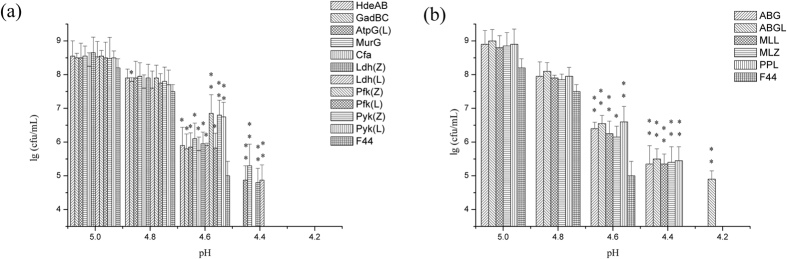
Effects of single acid-tolerant gene overexpression (**a**) and co-expression (**b**) on acid resistant capacity of strains. 100 μL of serially diluted cells suspension samples were spread in triplicate on seed media agar plates and incubated at 30 °C for 48 h. *p < 0.05, **p < 0.01, t-test.

At the same time, all engineered strains obtained higher survival rate in low pH condition (Fig. 5a,b). Strain F44 (ABGL) displayed the highest survival rate to low pH compared with the original strain F44 (Fig. 5b). The strong acid-tolerant capacity observed from the F44 (ABGL) should be the result of the overexpression of different proteins related to different mechanisms of acid tolerance. These data suggested that the higher nisin yield of the engineered strains than F44 is the result of a significant increase in acid tolerance capacity.

### Higher expression of acid tolerant genes ensures the stability of the intracellular pH

**Figure 6:**
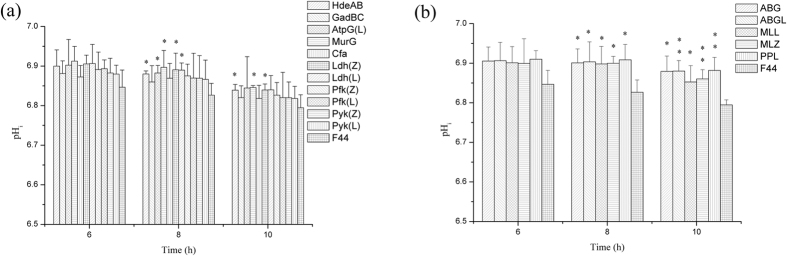
The intracellular pH of F44 and the engineered strains during fermentation process ranging at 6 h, 8 h and 10 h. (**a**) Single acid-tolerant gene over-expression strains; (**b**) Acid-tolerant genes co-overexpression strains. Samples were taken at every 2 h for 6-10 h. Error bars, SD from three independent experiments. *p < 0.05, **p < 0.01, t-test.

Intracellular pH (pH_i_) plays a major role in response to acid stress in lactic acid bacteria^44^,^45^. In order to further explore the influence of the acidic fermentation environment on the engineered strains and the original strain F44, the pH_i_ of these strains were measured during the fermentation process. Obvious differences in pH between F44 and the engineered strains were observed after 6, 8, and 10 h of fermentation. The engineered strains appeared to have the ability to maintain a higher pH_i_ than the F44 (Fig. 6a,b). This indicated that the engineered strains could avoid the sharp decrease of pH_i_ under acid stress effectively. Significant pH_i_ differences were detected in the engineered strains and the original strain F44 in response to acidic environment, which proved the protection of the acid tolerant genes on the *L. lactis* strain under acid environment. The data above showed that the increase of nisin yield might be the result of the stabilization of the intracellular pH by the overexpression of some acid tolerant genes.

### Fed-batch fermentation of the engineered strains helps to promote nisin yields

**Figure 7:**
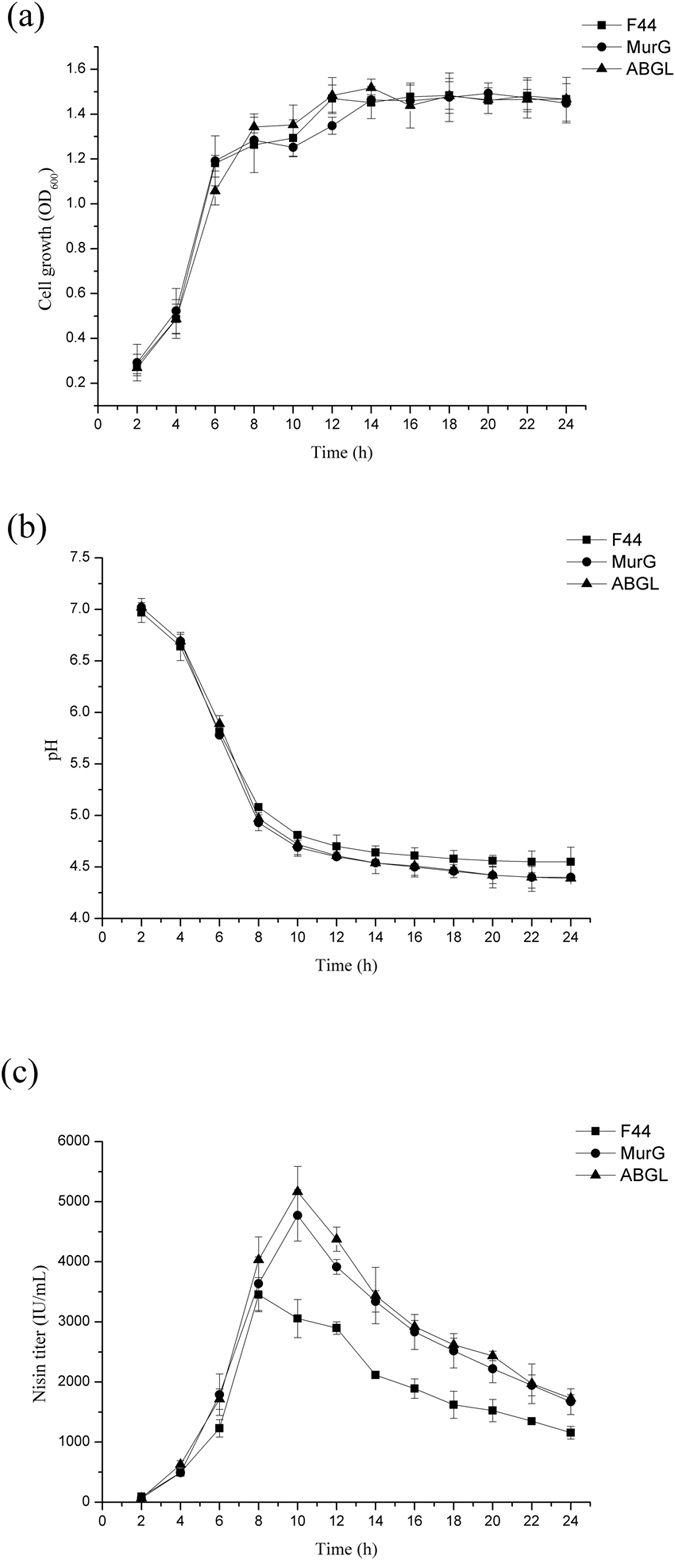
Fed-batch culture analysis of cell growth (**a**), pH of broth (**b**) and nisin production (**c**) with F44, transformants with single expression of *murG* and combinatorial expression of *hdeA*, *hdeB murG* and *ldh* genes, respectively. The culture temperature was maintained at 30 °C. Concentrated sucrose (500 g/L) was fed at a constant rate of 2 mL/h between 6–12 h during fermentation process. Samples were taken at every 2 h.

To further identify the performance of the artificial acid tolerant module, we performed fed-batch fermentation to optimize the fermentation processes. For F44, the consumption rate of sucrose was high in the initial 6 h, while the sucrose concentrations remained unchanged after 12 h^6^. The sucrose was added during fermentation process as an energy supplement for the cell growth and the nisin production. As shown in Fig. 7a,b, the growth rate of F44 was still higher than other engineered strains in the exponential phase. With the fermentation time extended, the biomass of acid tolerant strains were similar to that of F44. There were no significant differences on the biomass among F44 and other two strains, F44(ABGL) and F44 (MurG). Nisin production of F44 (ABGL) and F44 (MurG) was obviously higher than that of the original strain F44 (Fig. 7c). Compared with the nisin titer of F44, the nisin titer of F44 (MurG) and F44 (ABGL) were improved from 3454 IU/mL to 4774 IU/mL and 5167 IU/mL, increased by 38.2% and 49.6%, respectively. Although the growth conditions of the strains were similar, the pH_i_ of engineered strains should be significantly higher than that of F44 due to the overexpression of the acid tolerant genes which provided the relatively mild intracellular environment for cell growth and nisin production.

**Figure 8:**
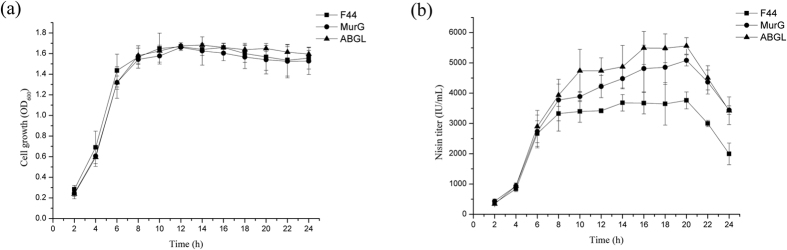
Effects of pH control on cell growth (**a**) and nisin production (**b**) during fed-batch fermentation process. The pH of culture was controlled at 6.0 for 6–24 h via the addition of NaOH (10 M).

To increase the nisin production and reduce the restriction of acid stress, the broth was maintained at pH 6.0 from 6^th^ hour by adding NaOH solution (10 M). All strains grew fast initially during the first 6 h. F44 grew faster than the engineered strains during the exponential phase, while the difference between F44 and other strains diminished by the fermentation extension (Fig. 8a). When pH was adjusted to 6.0, the nisin titer achieved the fairly high level and then increased slowly, finally dropped gradually. This indicates that the bacteria require an adaptive period when encountering environmental change. Then strains recovered rapidly, the cell growth continued and nisin was accumulated more. The nisin titer of strain F44 (ABGL) also reaches peak value of 5563 IU/mL which was 1.48 fold that of the original strain, and was also higher than that of F44 (MurG) during the whole fermentation process (Fig. 8b).

Similarly, we also measured the OD_600_ and nisin production through the fed-batch fermentation, of which the pH was adjusted to 5.5, 5.0, and 4.0, respectively (see Supplementary Fig. S7). In the culture at pH 5.5, the growth of all strains is only marginally affected by pH regulation, and the nisin yield had a quite similar trend at medium pH from 6.0 to 5.5 (see Supplementary Fig. S7a,b). The nisin titer of F44 (ABGL) was 4674 IU/mL, which increased by 24.2% than that of F44.

The low pH of broth has a great effect on the growth and production of nisin. The growth of all strains was strongly inhibited in acidic environment after adjusting the pH of fermentative broth (see Supplementary Fig. S7c,e). F44 grew faster during the exponential phase, while the difference between F44 and the engineered strains diminished in the condition of pH 5.0 and 4.0, which showed that overexpression of acid tolerant genes indeed protected cells under acid stress.

The activity of nisin is better at low pH, but the growth of all strains was restricted which lead to weakening the nisin production (see Supplementary Fig. S7d). When pH of medium was adjusted to 5.0, the nisin titer decreased at first and then transitorily increased in an adaptive buffer stage, finally dropped gradually. The nisin titer of F44 (ABGL), which appeared a short-term bounce of 3671 IU/mL after pH adjustment, was still significantly higher than F44, but that of strain F44 did only reach the level of 2799 IU/mL before acidification of broth. The nisin titer of engineered strains was always higher than that of F44 during the whole fermentation process. For the culture at pH 4.0 by adding HCl at 6 h, the final biomass levels further decreased. The growth retardation of all strains was observed after the acidifying operation, which illustrated that improving acid tolerance of engineered strains have reached the limitation, which has a great impact on nisin production (see Supplementary Fig. S7f).

## Discussion

Many metabolites produced in the industrial fermentation process could seriously inhibit the bacterial growth^46^, with the example of acidic metabolites, such as lactic acid and some carboxylic acids. These metabolites not only inhibit the cell growth but also reduce the yield of the target products. During the lactate fermentation process, the accumulation of lactic acid produced by lactic acid bacteria acidified the broth, therefore suppressed the cell growth of lactic acid bacteria whose optimal pH was about 6.3–6.9, and also limited the yield of lactic acid^47^. The traditional method to deal with this problem was to maintain the optimal pH of broth by adding alkali, which not only complicated the fermentation process but also might cause contamination^48^,^49^,^50^. It was reported that one kind of bacteria consuming lactic acid was co-cultured with *L. lactis*^51^, which could reduce the influence of lactic acid and pH on the growth of bacteria and nisin production. But the co-cultivation increased the difficulty of the industrial fermentation and the cost of the separation process. Introducing the acid tolerant genes to improve acid resistance of cells is an alternative method, by which, the strains can maintain normal growth rate in the late stationary stage of fermentation and therefore the accumulation of target metabolites can be promoted.

The pH value of broth can decrease to 4.5 due to the accumulation of acidic metabolites (lactic acid) during fermentation, which limits the normal growth of *L. lactis*, whose optimal pH is about 7.2, and then also affects the production of nisin. At the same time, the fluctuations of intracellular physiological environment under acid stress also lead to the reduction of physiological activity. The improvement of acid tolerance capacity is beneficial to prolong the fermentation period, and improve the growth activity of bacteria, which is of great importance to ferment process producing acidic metabolites. In response to acid stress, a large number of acid tolerant proteins were expressed to protect *L. lactis* cells. In recent years, the development of genome sequencing and various omics approaches have assisted to illuminate many acid tolerant mechanisms. Many genes were found significantly upregulated under acid stress by transcriptional analysis^52^. Some of these genes were further proved to be involved in various mechanisms under stress conditions^53^,^54^. Acid tolerant genes expression in the engineered strains could improve the acid tolerance capacity and maintain good cell growth, and therefore might improve nisin yield. We attempted to overexpress 17 acid-tolerant genes and 6 lactic acid synthetic genes in *L. lactis* F44 (Table 1). It was found that acid tolerance and nisin yield of the engineered strains could be improved in varying degree. Among these engineered strains, overexpression of *murG*, *hdeAB*, *ldh* makes better performance on the robustness and nisin production of cells.

Encoding a kind of glycosyltransferase, *murG* is involved in the peptidoglycan synthesis of bacterial cell walls by catalyzing the successive transfers of the N-acetylglucosamine moieties onto the carrier lipid^43^. The cell wall-associated protein MurG might be abundant and accumulated under acidic conditions. The expression of *murG* lead to higher survival, and the enzyme might act as a key role of fixing cell walls under acid stress. HdeA and its structural homologue HdeB could maintain the optimal chaperones activity at pH 2.0 and pH 4.0, respectively, therefore could be used as acid-resistance proteins in bacteria^55^. These two chaperones can help bacteria to survive in acidic protein-unfolding conditions. It is interesting that the chaperones HdeA and HdeB from *E. coli* could take effect in *L. lactis* which belongs to gram-positive bacteria without periplasmic space.

Besides, during the production of lactic acid, as one end product of the glucose metabolism in the lactic acid bacteria, energy can be produced in this process. As the key enzymes involved in the synthesis of lactic acid, *pyk*, *pfy* and *ldh* were confirmed to a higher expression level in lactate fermentation by proteomic analysis^52^,^56^. Most acid tolerant mechanisms need to consume energy. Under low pH conditions, the energy from sugar metabolism is mainly used to withstand harsh environment, therefore the cell growth and the production capacity are affected by the energy supply. So enhancing the lactic acid production and energy generation by overexpressing these genes could effectively improve the cell growth, acid tolerance capacity and nisin yield.

It is interesting that the optical density of F44 was higher than that of engineered strains which may result from the faster growth rate of F44 in the exponential phase (see Supplementary Table S3). Indeed, the replication and expression of acid tolerant genes from the vector might cause metabolic burdens in the engineered strains and therefore delayed their growth. After 10 h fermentation, the cell growth and the nisin production of all strains were in stagnation stage with pH close to about 4.5 (see Supplementary Table S3). At the same time, the nisin accumulation dropped greatly, and nisin titer of all strains decreased in a same trend, which illustrated that improving acid tolerance of engineered strains has reached the limitation (see Supplementary Fig. S2c and Table S3). These results showed it was difficult to maintain physiological activities of cells as well as normal metabolic process under severe acid stress. However, as expected, nisin yields of the engineered strains were higher than that of the original strain F44, especially in late fermentation stage, which meant these cells could be resistant to acidic environment and remained active and stable (Fig. 2). Although the nisin yields of engineered strains and F44 exist obvious differences, there were no significant differences in the expression levels of nisin gene cluster between them according to the results of qRT-PCR. Thus, it is concluded that improvement of nisin yield is resulted from that overexpression of acid tolerant genes makes the strains more robust and adaptive under acid stress rather than increases the expression of genes related to nisin biosynthesis. The results of combined overexpression of *hdeAB*, *murG*, and *ldh*(Z) in F44 showed nisin yield of the engineered strain was improved further, and better effects were also achieved by analysis of fed-batch process (Figs 7b and 8b). Although the nisin yield at pH 5.5 did not reach a higher level, the value of 5.5 seemed to be the limit culture pH value enabling growth to proceed, and the inhibition of low pH (pH 5.0 and 4.0) on the growth and the ability of nisin production was obvious (see Supplementary Fig. S7). Neither the ΔpH between the cytoplasm and the culture medium nor the pH_i_ were maintained constant when the culture pH value was lower than 5.0, and the efficiency of biomass synthesis relying on the energy supply also decreased^57^. The lower the pH was, the less the cell concentration was obtained when the strains reached the stationary phase, and the nisin production dropped greatly. The nisin titer of all strains decreased in a general trend, which showed it was difficult to maintain physiological activities of cells as well as normal metabolic process due to acidic damage during the cultures performed at medium pH 5.0 or lower. Although the nisin titer of the engineered strains harboring several acid tolerant genes was higher than the single gene expression strains, the overexpression of more acid tolerance genes would consume more energy, therefore, the overload of acid tolerant genes overexpression might affect the higher improvement of the nisin titer. In addition, from the results of acid stress assay, compared with the original strain, the engineered strains constructed in this paper had more survival rates in the environment of lower pH, and also had higher nisin yield, which confirmed our hypothesis that overexpressing these genes would increase the production of nisin (Fig. 5a,b).

Maintaining relatively stable intracellular pH is important for microorganisms, which can ensure the normal physiological behavior of cells and guarantee the activity of enzymes^58^,^59^. Intracellular pH should be affected by the large changes of environmental pH. Exceeding certain range of environmental pH fluctuation would influence the dynamic balance of the intracellular physiological activities and even affect the survival of cells. The engineered strains showed higher pH_i_ value than that of the original strain (Fig. 6a,b). These engineered strains could maintain a relatively stable pH_i_ and enhance the adaptability of the bacteria in certain acidic environment.

According to what we learnt, it is the first report that the nisin production was improved by over-expressing acid-tolerant genes, which would provide some clues for the construction of other acid-tolerant microorganism. And in the future study, we want to optimize the promoters to regulate the expression level of the genes and reduce the side effects resulted from the excessive expression of heterogenous proteins. In addition, we also plan to construct an acid-tolerant network which can regulate the engineered strains in multi-level and cross joint manner.

## Methods

### Bacteria strains and growth conditions

The bacterial strains and plasmids used in the study are listed in the Supplementary Table S1. The *L. lactis* strain F44 was used for the phenotypic examination throughout this study. The *E. coli* TG1 was used for plasmid preparation. All the *E. coli* strains were grown at 37 °C, with shaking at 180 rpm in the Luria-Bertani (LB) medium. All *L. lactis* strains were preserved and cultured in seed medium (wt/vol) containing peptone (1.5%), yeast extract (1.5%), sucrose (1.5%), KH_2_PO_4_ (2.0%), NaCl (0.15%), corn steep liquor (0.3%), cysteine (0.26%), and MgSO_4_·7 H_2_O (0.015%). The pH value was adjusted to 7.2 with 10 *M* NaOH before autoclaving at 121 °C for 20 min. The antibiotics erythromycin (Em^r^) (100 μg/mL for *E.coli* TG1, 5 μg/mL for *L. lactis* F44) was used for selection. The tolerance agar plates were supplemented with 1.5% agar after autoclaving. *Micrococcus flavus* ATCC 10240, preserved in the laboratory, was used as an indicator strain for the bioassay of nisin.

### Nisin activity assay

A stock solution of nisin was prepared by mixing 0.1 g of nisin standards (Sigma, USA) in 10 mL of 0.02 *M* HCl (10^6^ IU/mL) and boiling for 5 min. The stock solution was diluted using 0.02 *M* HCl to standard nisin solutions. Nisin standards were added into the autoclaved initial fermentation medium at pH 2.0, 3.0, 4.0, 5.0, 6.0 and 7.0, respectively, and boiling for 5 min. All the fermentation media containing 4000 U/mL nisin (1 g of nisin in 100 mL media) were incubated at 30 °C. The fermentation broth was sampled every 2 h for nisin titer analysis. 500 μL of fermentation broth was mixed with the same volume of 0.02 *M* HCl. The mixture was boiled for 5 min and then appropriately diluted with 0.02 *M* HCl. 1.5% (vol/vol) tween 80 (JiangTian, Tianjin, China) (tween 80 enhances nisin diffusion into the agar medium) and 1% (vol/vol) and 1% (vol/vol) indicator strain *M. flavus* ATCC 10240 buffer (the final concentration was 10^7^ cfu/mL) was poured into the 26-mL assay medium which was cooled to about 45 °C after autoclaving. The medium was poured into a sterile plate for solidification and precultivation. Standard nisin solutions and test solutions were infused into individual wells (80 μL per well) which had been bored into the assay plate (8 wells per plate) using a 7-mm-diameter metal tube, and then the plates were incubated at 37 °C for 24 h. Zones of inhibition were measured and the regression equation was calculated. Each assay of standard sample or the broth sample was performed in triplicate.

### Fermentation performance at acidic condition

The seed medium was inoculated with bacterial colonies and incubated overnight. 5% of the overnight culture broth was used to inoculate fermentation medium for 12 h at 30 °C. The initial pH of fermentation medium were adjusted to 2.0, 3.0, 4.0, 5.0, 6.0 and 7.0, respectively. Cell density (OD_600_), pH values, and nisin titer of broth samples were measured every 2 h.

### DNA manipulations and genetic construction

The primers of acid tolerant genes used in the study which were designed by primer premier 5 (Premier, Canada) are listed in Supplementary Table S2. These genes were directly amplified from *Lactobacillus casei* Zhang, *L. lactis* subsp. *lactis* F44 or *E. coli* DH5α via polymerase chain reaction (PCR). The restriction enzyme cutting sites were simultaneously inserted into the amplified gene. Combinations of two, three or more genes were constructed via overlap extension PCR. The resulting fragments were digested with BamHI and HindIII (or SmaI), and then ligated into plasmid pLEB124, cut with BamHI and HindIII (or SmaI) to generate the resulting plasmids. The resulting plasmids were transformed into *E.coli* TG1 by heat shock transformation for enrichment^60^. After antibiotics selection, the plasmids were extracted with TIANprep Mini Plasmid Kit (TIANGEN, China), and then were transformed into the *L. lactis* F44 by electroporation transformation^61^.

### Nisin titer assay

The nisin titer assay was described previously^6^. Briefly, the nisin standards (Sigma, USA) and the fermentation broth which have removed the cells by centrifugation at 8000 rpm for 5 min, were boiled for 5 min. Then these samples were serially diluted with 0.02 M HCl. After autoclaving, the 26-mL cooled assay medium was inoculated with 1% (vol/vol) *M. flavus* buffer, with the concentration of 10^7^ cfu/mL. Then the medium was added with 1.5% (vol/vol) tween 80 (JiangTian, Tianjin, China) to enhance nisin diffusion. The mixed medium was quickly poured into sterile plates. After solidification and pre-cultivation, we used a 7-mm-diameter metal tube to drill into the assay plate (8 wells per plate) and removed the agar from the well. Standard nisin solutions and broth solutions were injected into the respective wells (80 μL per well) and the plates were incubated at 37 °C for 24 h. Inhibition zones were measured by calipers. A regression equation was calculated from the measured data. Each assay of the samples including standards and broth was performed in triplicate.

### RNA isolation and transcriptional analysis by quantitative real-time PCR

*L. lactis* strain F44 genome was isolated with the TIANamp Bacteria DNA Kit (TIANGEN). The genome was diluted to concentrations of 10^−5^, 10^−4^, 10^−3^, 10^−2^, 10^−1^. After acid shock at pH 7.0, 6.0, 5.0 or 4.0 respectively, total RNAs were isolated with ZR RNA MiniPrep (The Epigenetics company). For qRT-PCR, 0.1~0.5 μg of total RNA were reverse transcribed together with 1 μL corresponding primers in a total reaction volume of 12 μL using TIANScript RT Kit (TIANGEN). The reaction system was harvested by 3000 rpm for 30 s. Each reaction was incubated at 65 °C for 5 min, followed by incubating on ice. Then the reactions were run at 40 °C for 60 min, followed by 70 °C for 5 min to terminate the complementary DNAs synthesis. The generated cDNAs were stored at −70 °C for further qRT-PCR. qRT-PCR was performed with Power SYBR Green PCR Master Mix (Applied Biosystems). Briefly, a 50 μL-reaction solution containing 10~100 ng of cDNA, 200 nM each primer (see Supplementary Table S2), 25 μL 2 × Ultra SYBR Mixture (with ROX) and sterile water was analyzed on a LightCycler 480 Real-Time PCR System (Roche, Switzerland) according to the manufacturer’s instructions. Reactions were run in triplicate in three independent experiments for each condition. qRT-PCR conditions were as follows: 1 cycle at 95 °C for 10 min, 40 cycles of denaturation at 95 °C for 10 s, annealing at 55 °C for 10 s and extension at 72 °C for 20 s. The 16S rRNA gene was used as an internal control to normalize cycle threshold (C_T_) values. Difference in the relative expression levels were calculated with 2^−(ΔΔC_T_) method. We performed a 10-fold dilution series experiment using the target assay to establish the standard curve, and the slope of the standard curve can be translated into an efficiency value: PCR efficiency = 10^−1/slope^–1^62^.

### Measurement of intracellular pH (pH_i_)

The pH_i_ was measured with the fluorescence method using 5 (and 6-)-carboxyfluorescein succimidyl ester as the fluorescent probe^63^. Calibration curves were established to exclude artificial bias by different environmental conditions and cell states. pH_i_ values were determined by the radio of the fluorescence signal measurements at excitation wavelengths of 490 nm (pH-sensitive wavelength) and 440 nm (pH-insensitive wavelength) with Fluorescence Spectrophotometer F-2700 (Hitachi High-Tech, Japan). Loading of cells with the fluorescent probe, the establishment of calibration curves and the determination of pH_i_ all followed the procedure which Breeuwer *et al*. described previously^63^.

### Acid tolerance capacity assay

To investigate the acid tolerance, the wild type strain and the recombination strain after activation culture overnight at 30 °C in the seed medium were harvested in mid-exponential growth phase. Aliquots of cells suspension were diluted to concentrations of 10^−6^, 10^−5^, 10^−4^, 10^−3^ and 10^−2^. Cell survival numbers were estimated, where 100 μL of serially diluted samples were spread in triplicate on seed media agar plates with different pH values 4.0, 4.2, 4.4, 4.6, 4.8 and 5.0, and incubated at 30 °C for 48 h. Plates with colonies in the range of 30 to 300 were then used to calculate the average number of CFU/ml.

### Fermentation in flasks

The original strain F44 and the engineered strains expressing the acid tolerant genes were inoculated in the seed medium for overnight after the cultivation on seed plates at 30 °C for 48 h. The flask fermentation experiments were carried out in 250-mL Erlenmeyer flasks containing 100 mL of fermentation medium with peptone (1.5%), yeast extract (1.5%), sucrose (1.5%), KH_2_PO_4_ (2.0%), NaCl (0.15%), corn steep liquor (0.3%), cysteine (0.26%), and MgSO_4_·7 H_2_O (0.015%). Five milliliters of the overnight cultures were inoculated in triplicate into the static flasks, and incubated for 14 h at 30 °C. Samples were withdrawn every 2 h for cell density analysis, fermentation broth pH, intracellular pH (pH_i_), lactic acid concentration, and nisin production.

### Fed-batch fermentation

The fed-batch fermentation experiment was conducted at 30 °C for 24 h. Initially the fermentation medium was adjusted to pH 7.2 with 10 *M* NaOH as described above. The medium was inoculated with 5% of the seed culture. After the fermentation of 6 h, the pH of fermentation broth was controlled at 6.0 by the addition of 10 *M* NaOH. 3 mL sucrose solution (500 g/L) was added to the fermentation broth at 6 h, 8 h, 10 h and 12 h. The fermentation broth was sampled every 2 h for cell density analysis, lactic acid concentration, residual sugar concentration, and nisin production.

### Statistical analysis

Optical density (OD) was measured at 600 nm with TU-1810 spectrophotometer to monitor the *L. lactis* cell growth. The pH values of the fermentation broth were measured with FE20 benchtop pH meter (Mettler Toledo, Swiss). To measure the lactic acid concentration and sucrose concentration during the fermentation process, samples were periodically collected for estimating lactate and sucrose concentration in supernatants after removing the cells by centrifugation (8000 rpm, 5 min, 4 °C). The concentration of the residual sugars in the broth was assayed by the dinitrosalicylic acid reagent (DNS) method^64^. The lactate concentration was quantified by a biosensor SBA-90 (Biology Institute of Shandong Academy of Sciences, China)^65^. To assess the statistical significance of differences in resistance to acid stress, student’s t tests were used. For the significance of gene expression differences, the statistical t-test was used to identify genes differentially expressed between the wild type strain and the engineered strain. Statistical significance of nisin titer and pH_i_ was estimated using t-test. Statistical analyses of the data were performed using SPSS software version 19.0 (IBM, USA).

## Additional Information

**How to cite this article**: Zhang, J. *et al*. Enhance nisin yield via improving acid-tolerant capability of *Lactococcus lactis* F44. *Sci. Rep.* **6**, 27973; doi: 10.1038/srep27973 (2016).

## Supplementary Material

Supplementary Information

## Figures and Tables

**Table 1:** Summary of the information about acid tolerance genes and lactic acid synthetic genes. *L. lactis* F44 was actually obtained by genome shuffling of *L. lactis* YF11 (CGMCC NO. 7.52).

Gene ID	Gene	Organism	Function
**Obstacle of extracellular H**^**+**^			
–	*murA*	*Lactococcus lactis* F44	UDP-N-acetylglucosamine 1-carboxyvinyltransferase
–	*murG*	*Lactococcus lactis* F44	UDP-N-acetylglucosamine–N-acetylmuramyl-(pentapeptide) pyrophosphoryl-undecaprenol N-acetylglucosamine transferase
gi|944811	*cfa*	*Escherichia coli* DH5α	Cyclopropane-fatty-acyl-phospholipid synthase
**Consumption of intracellular H**^**+**^			
–	*mleS*	*Lactococcus lactis* F44	Malolactic enzyme
–	*mleP*	*Lactococcus lactis* F44	Malate/lactate antiporter
–	*gadB*	*Lactococcus lactis* F44	Glutamate decarboxylase
–	*gadC*	*Lactococcus lactis* F44	Glutamate/gamma-aminobutyrate antiporter
–	*gadBC*	*Lactococcus lactis* F44	Glutamate decarboxylation system
gi|948643	*cadA*	*Escherichia coli* DH5α	Lysine decarboxylase, inducible
gi|948654	*cadB*	*Escherichia coli* DH5α	Probable cadaverine/lysine antiporter
**Exportation of intracellular H**^**+**^			
–	*atpD*	*Lactococcus lactis* F44	ATP synthase subunit beta
gi|948244	*atpD*	*Escherichia coli* DH5α	ATP synthase subunit beta
–	*atpG*	*Lactococcus lactis* F44	ATP synthase gamma chain
gi|948243	*atpG*	*Escherichia coli* DH5α	ATP synthase gamma chain
**Proteins refolding**			
gi|948025	*hdeA*	*Escherichia coli* DH5α	Acid stress chaperone HdeA
gi|948026	*hdeB*	*Escherichia coli* DH5α	Acid stress chaperone HdeB
–	*hdeAB*	*Escherichia coli* DH5α	Periplasmic acid stress protein
**Alternation of carbohydrate metabolic pathway**			
–	*pfk*	*Lactococcus lactis* F44	6-phosphofructokinase
gi|9459266	*pfk*	*Lactobacillus casei* Zhang	6-phosphofructokinase
–	*pyk*	*Lactococcus lactis* F44	pyruvate kinase
gi|9459267	*pyk*	*Lactobacillus casei* Zhang	pyruvate kinase
–	*ldh*	*Lactococcus lactis* F44	L-lactate dehydrogenase
gi|9460421	*ldh*	*Lactobacillus casei* Zhang	L-lactate dehydrogenase
