# Supplementary material for: Enhance nisin yield via improving acid-tolerant capability of Lactococcus lactis F44
Source: Sci Rep. 2016 Jun 16;6:27973. doi: 10.1038/srep27973 (PMC4910042; doi:10.1038/srep27973)
Supplement: Supplementary Information [file srep27973-s1.doc]

Supplementary information for

**Enhance nisin yield via improving acid-tolerant capability of *Lactococcus lactis* F44**

**Jian Zhang^1, 2, 3, +^ , Qinggele Caiyin^2, 3, +^ , Wenjing Feng^1, 2, 3, +^，Xiuli Zhao^1, 2, 3^，Bin Qiao^2, 3^,, , Guangrong Zhao^2, 3^,, Jianjun Qiao^1, 2, 3, *^**

^1^ Department of Pharmaceutical Engineering, School of Chemical Engineering and Technology, Tianjin University, Tianjin 300072, China.

^2^ Key Laboratory of Systems Bioengineering, Ministry of Education Tianjin, 300072,

China

^3^ SynBio Research Platform, Collaborative Innovation Center of Chemical Science

and Engineering, Tianjin 300072, China

**^*^** Correspondence and requests for materials should be addressed to J.Q. (e-mail: [jianjunq@tju.edu.cn](mailto:jianjunq@tju.edu.cn))

^+^ Jian Zhang, Qinggele Caiyin and Wenjing Feng contributed equally to this work.

**This file includes:**

Supplementary Figures 1–7;

Supplementary Tables 1–3;

Supplementary References.

**Supplementary Figures**


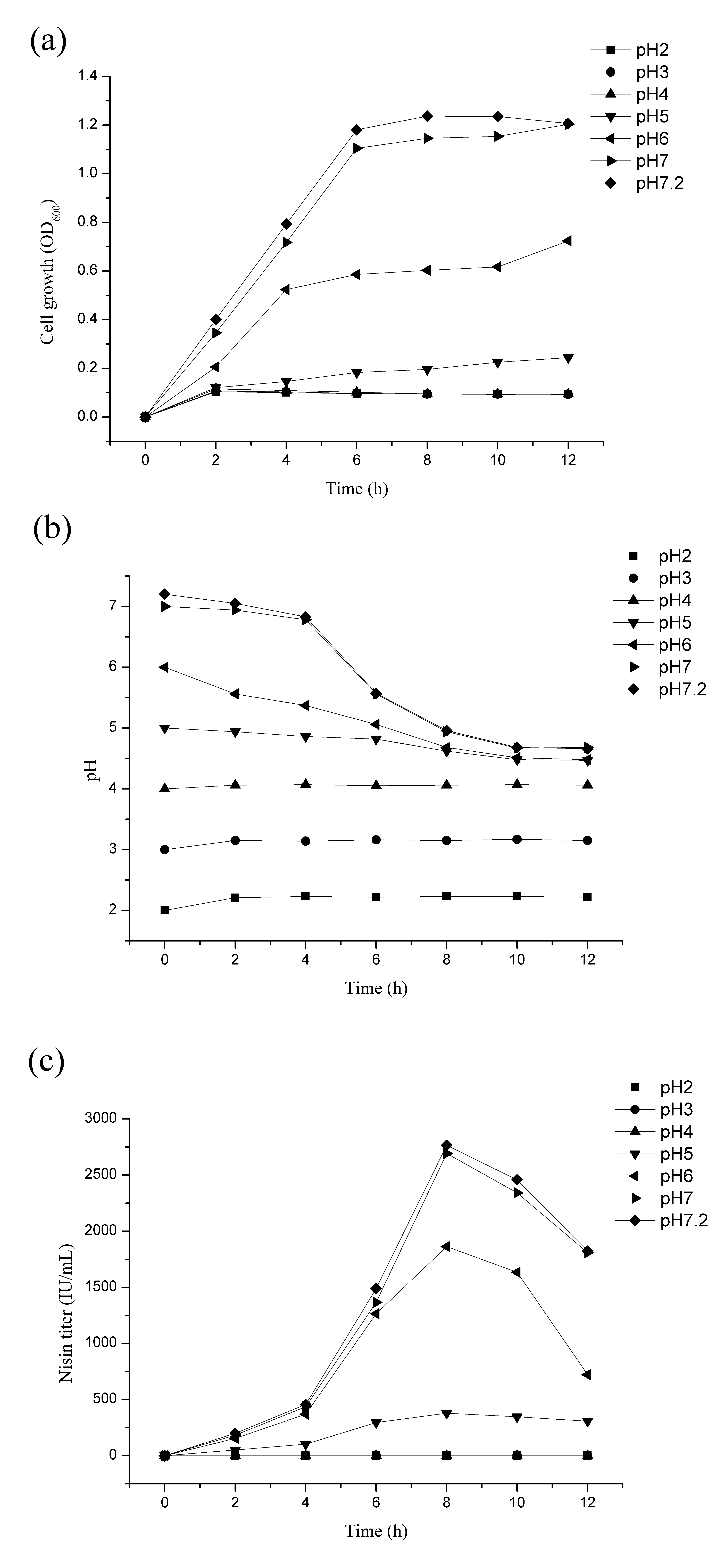


**Figure S1. The effect of different initial pH value of the fermentation medium（a:OD_600_；b: pH；c: nisin titer）**


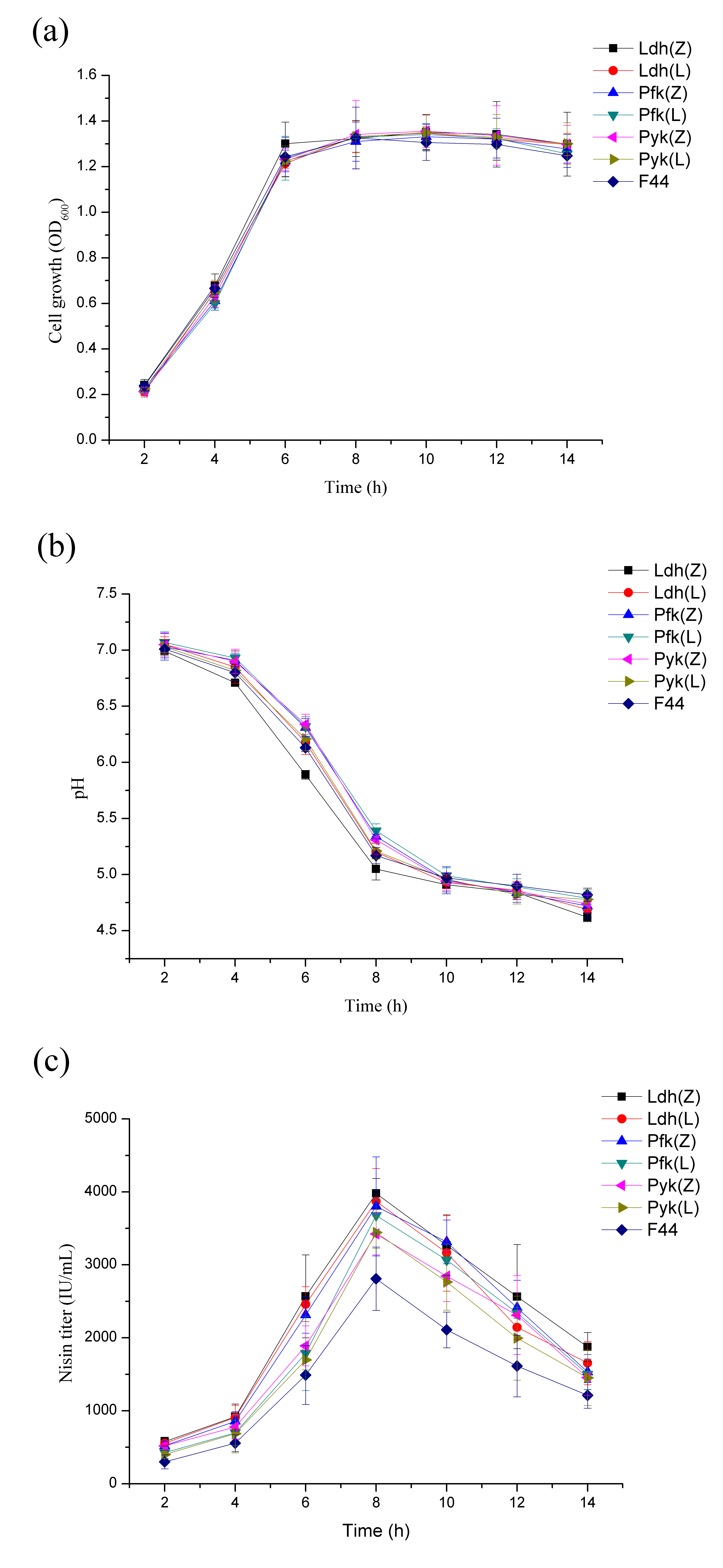


**Figure S2. Effect of different lactic acid biosynthesis genes on biomass of strains (a), pH of the fermentation broth (b) and nisin titer (c).**

**
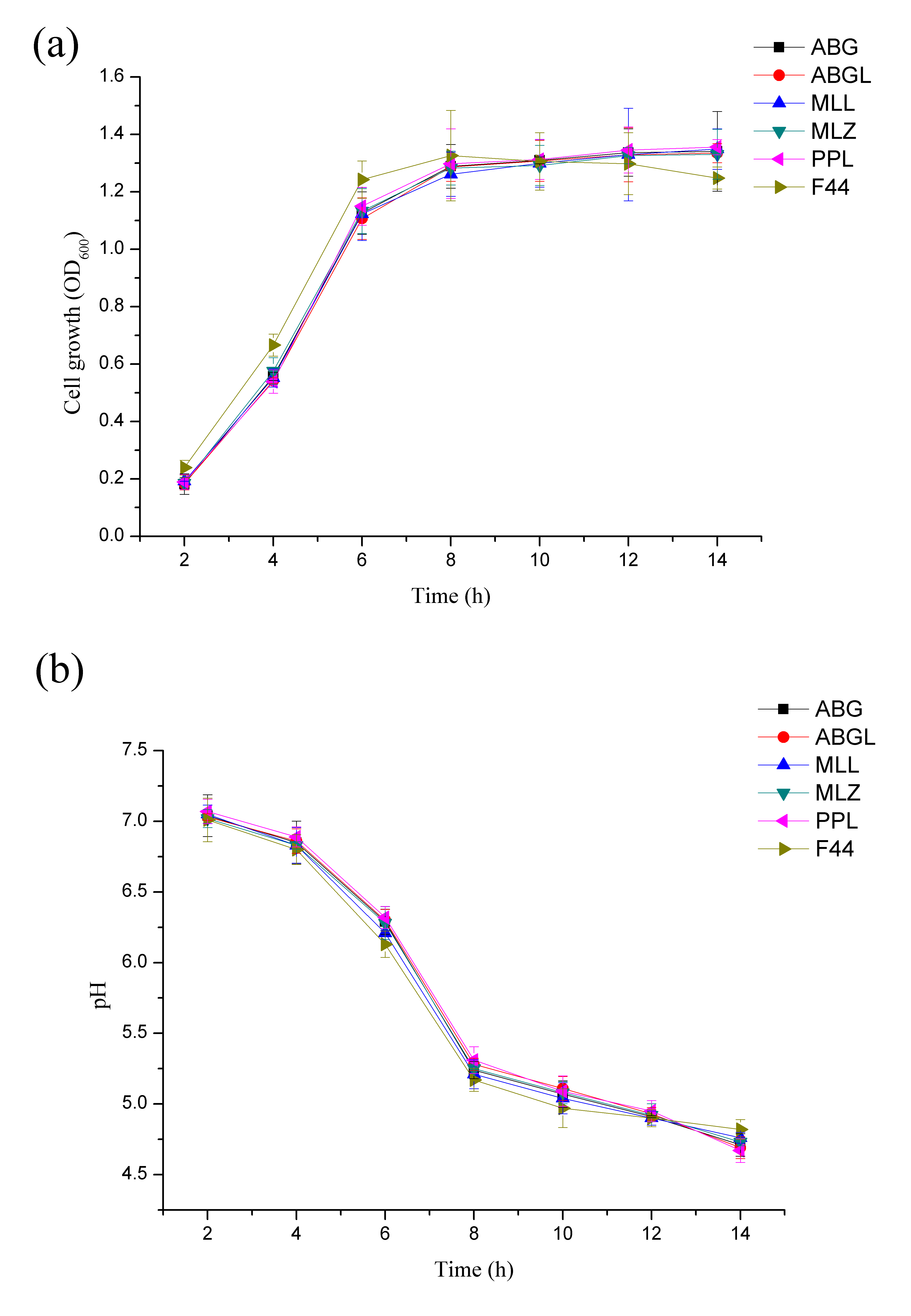
**

**Figure S3. Effect of several acid tolerance genes on biomass of strains (a) and pH of the fermentation broth (b).**

**
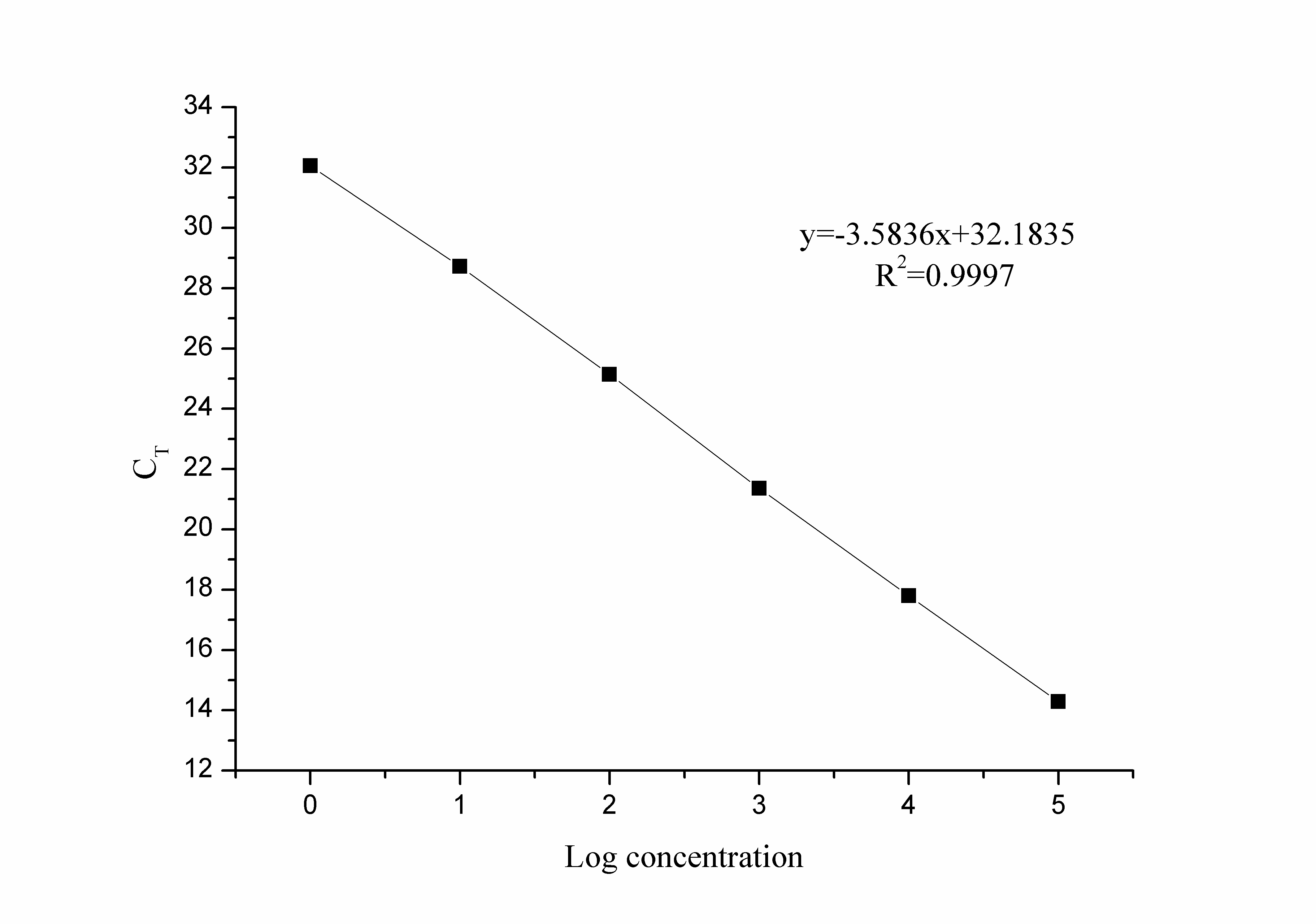
**

**Figure S4. The standard curve of qRT-PCR by performing a 10-fold dilution series experiment using the target assay.**


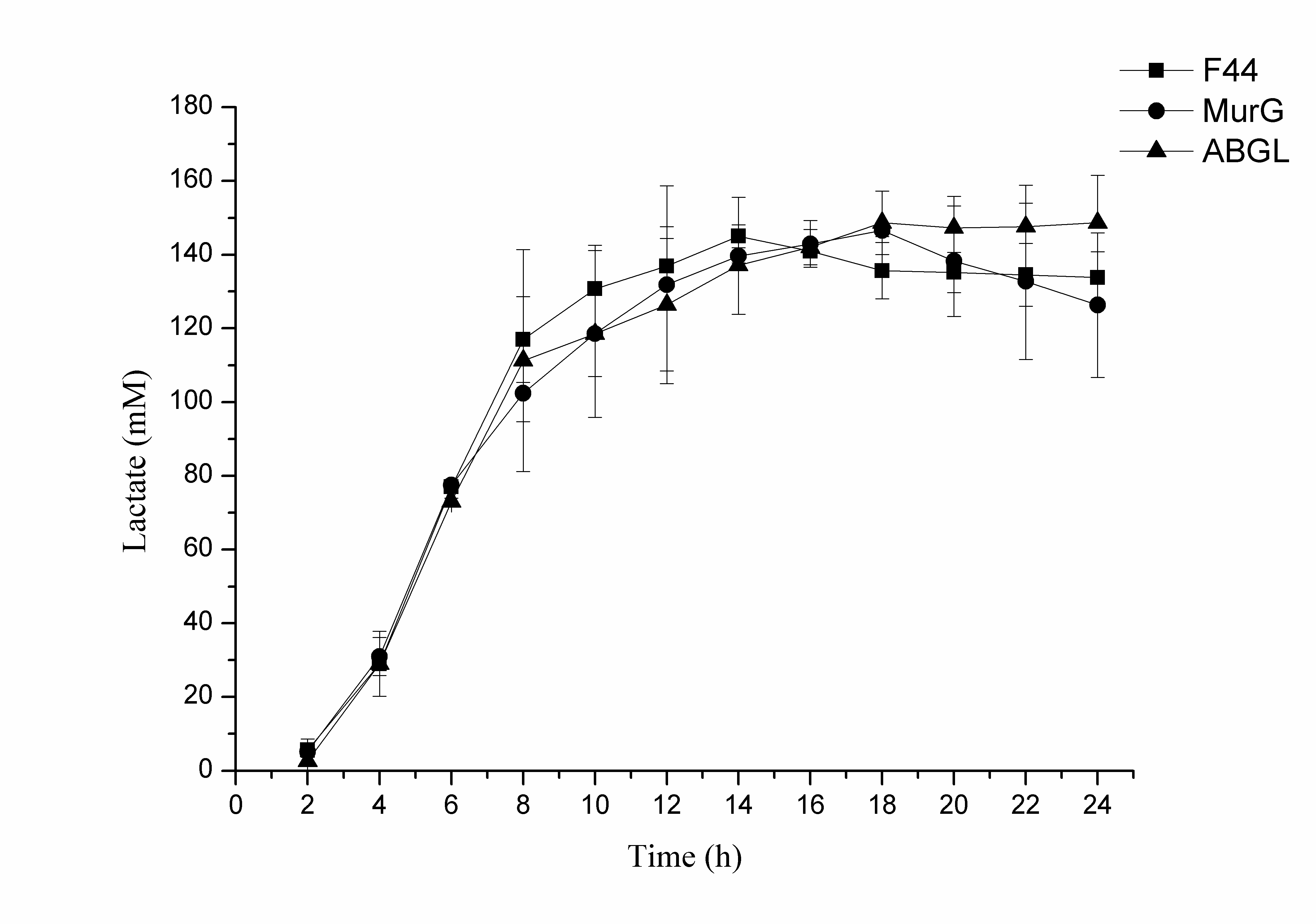


**Figure S5. Fed-batch culture analysis for lactate production with different strains.**

**
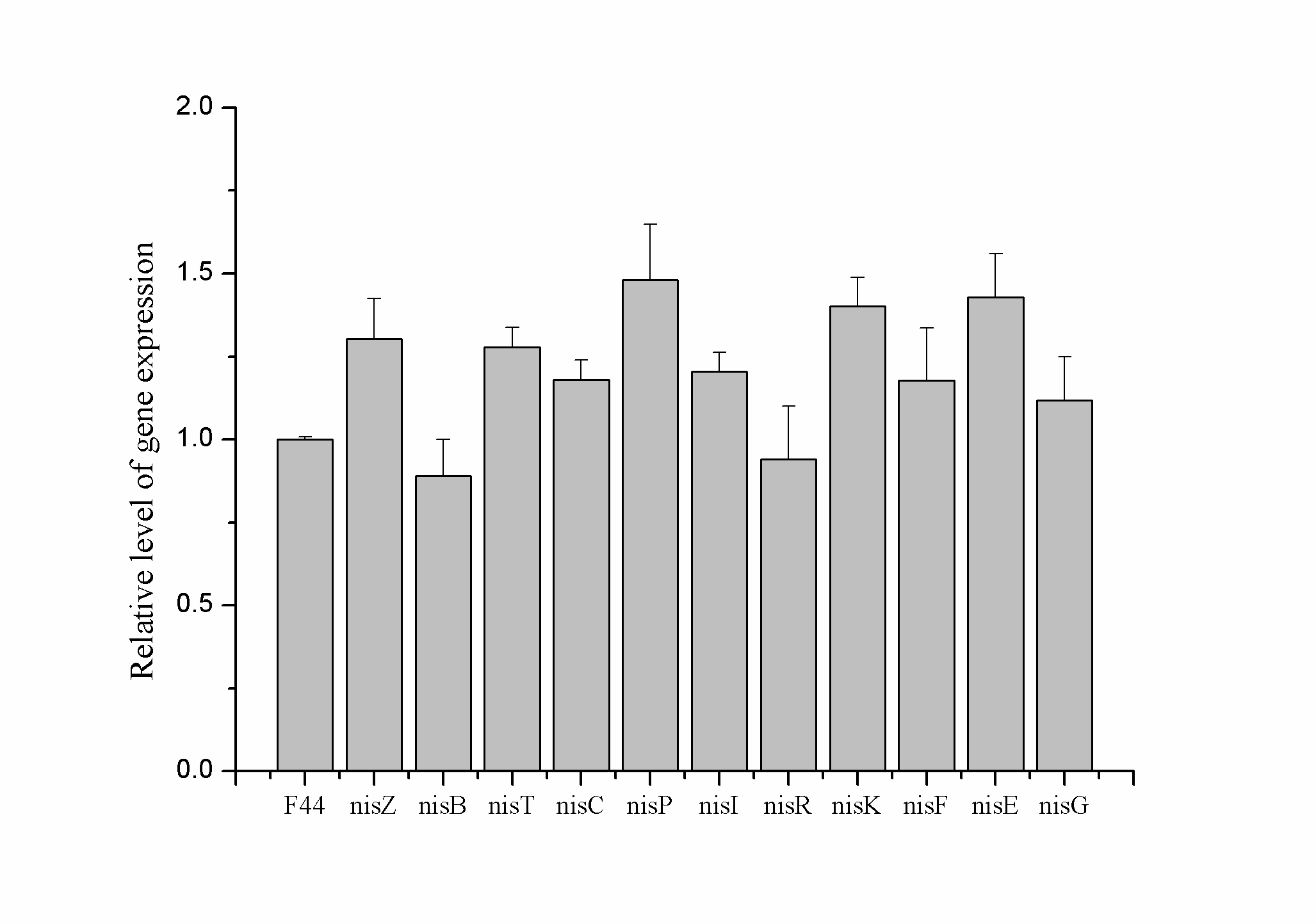
**

**Figure S6. The transcriptional analysis of nisin gene cluster in wild type F44 and the engineered strain F44 (ABGL) by qRT-PCR.**

**
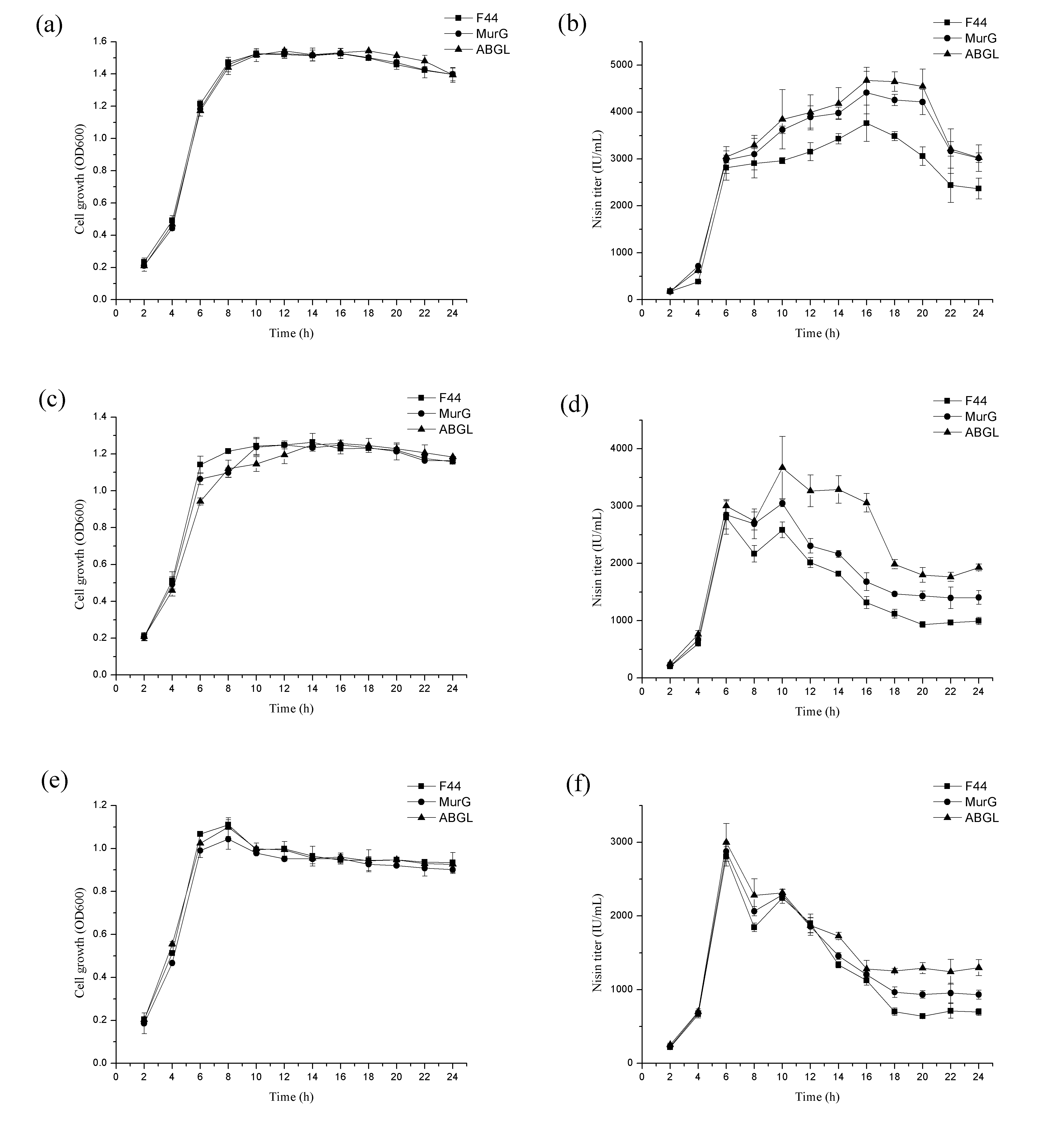
**

**Figure S7. Effects of pH control on cell growth and nisin production during fed-batch fermentation process (pH 5.5: (a) and (b), pH 5.0: (c) and (d), pH 4.0: (e) and (f)).**

**Supplementary Tables**

**Table S1. Bacteria strains and plasmids**

| **Name** | **Soure** | **Relevant characteristics** |
| --- | --- | --- |
| *Lactococcus lactis* F44 |  | Evolved from *Lactococcus lactis* YF11, nisin productor [1] |
| *Escherichia coli* DH5α |  | Laboratory stock |
| *Lactobacillus casei* Zhang |  | [2] |
| *Escherichia coli* TG1 |  | Laboratory stock |
| *Micrococcus flavus* |  | Laboratory stock |
| pLEB124 |  | Lactococal plasmid, Em^r^ |
| MleS | This study | pLEB124 derivative bearing a fragment of *mleS* gene, Em^r^ |
| MleP | This study | pLEB124 derivative bearing a fragment of *mleP* gene, Em^r^ |
| CadB | This study | pLEB124 derivative bearing a fragment of *cadB* gene, Em^r^ |
| CadA | This study | pLEB124 derivative bearing a fragment of *cadA* gene, Em^r^ |
| HdeA | This study | pLEB124 derivative bearing a fragment of *hdeA* gene, Em^r^ |
| HdeB | This study | pLEB124 derivative bearing a fragment of *hdeB* gene, Em^r^ |
| HdeAB | This study | pLEB124 derivative bearing a fragment of *hdeA-hdeB* genes, Em^r^ |
| Cfa | This study | pLEB124 derivative bearing a fragment of *cfa* gene, Em^r^ |
| GadB | This study | pLEB124 derivative bearing a fragment of *gadB* gene, Em^r^ |
| GadC | This study | pLEB124 derivative bearing a fragment of *gadC* gene, Em^r^ |
| AtpD (L) | This study | pLEB124 derivative bearing a fragment of *atpD* gene, Em^r^ |
| AtpD(E) | This study | pLEB124 derivative bearing a fragment of *atpD* gene, Em^r^ |
| AtpG(E) | This study | pLEB124 derivative bearing a fragment of *atpG* gene, Em^r^ |
| AtpG(L) | This study | pLEB124 derivative bearing a fragment of *atpG* gene, Em^r^ |
| MurA(L) | This study | pLEB124 derivative bearing a fragment of *murA* f gene, Em^r^ |
| MurG(L) | This study | pLEB124 derivative bearing a fragment of *murG* gene, Em^r^ |
| Ldh(Z) | This study | pLEB124 derivative bearing a fragment of *ldh* gene, Em^r^ |
| Ldh(L) | This study | pLEB124 derivative bearing a fragment of *ldh* gene, Em^r^ |
| Pfk(L) | This study | pLEB124 derivative bearing a fragment of *pfk* gene, Em^r^ |
| Pfk(Z) | This study | pLEB124 derivative bearing a fragment of *pfk* gene, Em^r^ |
| Pyk(L) | This study | pLEB124 derivative bearing a fragment of *pyk* gene, Em^r^ |
| Pyk(Z) | This study | pLEB124 derivative bearing a fragment of *pyk* gene, Em^r^ |
| GadBC | This study | pLEB124 derivative bearing a fragment of *gadB*-*gadC* genes, Em^r^ |
| ABG | This study | pLEB124 derivative bearing a fragment of *hdeA*-*hdeB*-*murG* genes, Em^r^ |
| ABGL | This study | pLEB124 derivative bearing a fragment of *hdeA*-*hdeB*-*murG*-*ldh* genes, Em^r^ |

Table S2. Oligonucleotide sequences

| Primer name | Primer sequence (5 to 3’) | Function | Source |
| --- | --- | --- | --- |
| MleS-F | CCCAAGCTT TGGAGGTTGTACGATGCG, *Hin*dIII | Cloning of *mleS* expression | *L. lactis* F44 |
| MleS-R | CGGGATCCTTAAGATATTCCCCTTAGTACTCTG, *Bam*HI |
| MleP-F | CCCAAGCTTGTACTAAGGGGAATATCTTAAAT, *Hin*dIII | Cloning of *mleP* expression | *L. lactis* F44 |
| MleP-R | CG GGATCC TATTTTAATAAAAGAATCGGAT, *Bam*HI |
| CadB-F | CCCAAGCTTTGAAATTAGGAGAAGAGCATGAGT, *Hin*dIII | Cloning of *cadB* expression | *E. coli* DH5α |
| CadB-R | CGGGATCCGAAAGGAGGAGCCTCGGA, *Bam*HI |
| CadA-F | CCCAAGCTTTTTTTACCTGGAGATATGACTATG, *Hin*dIII | Cloning of *cadA* expression | *E. coli* DH5α |
| CadA-R | CGGGATCCTTCCCTTGTACGAGCTAATTAT, *Bam*HI |
| A&B-F | CCCAAGCTTATGCATCATCACCATCACCACGAGGATATTACGATGAAAAAAGT, *Hin*dIII | Cloning of *hdeAB* expression | *E. coli* DH5α |
| A&B-R | CGGGATCCTGGCTCAACTTGCTACTCC, *Bam*HI |
| HdeA-F | CCCAAGCTTATGCATCATCACCATCACCACGTTGAGGATATTACGATGAAAAA, *Hin*dIII | Cloning of *hdeA* expression | *E. coli* DH5α |
| HdeA-R | CGGGATCCCTGTTCAATATGCAAGGAAGTAC, *Bam*HI |
| HdeB-F | CCCAAGCTTATGCATCATCACCATCACCACTGAATGGGTTACAAATATGAATAT, *Hin*dIII | Cloning of *hdeB* expression | *E. coli* DH5α |
| HdeB-R | CGGGATCCAGATGGCTCAACTTGCTACTC, *Bam*HI |
| Newgeneral F | GTTTGAAACGTATTACTGAAGGGAA | sequencing | pLEB124 |
| Newgeneral R | ATTCATTCTGCTAACCAGTAAGGC |
| Cfa-F | CCCAAGCTTATGCATCATCACCATCACCACGTTAGGAAAGGGAAGAATAACT, *Hin*dIII | Cloning of *cfa* expression | *E. coli* DH5α |
| Cfa-R | CGGGATCCCAACTTATCAATTGTCAAACTTAA, *Bam*HI |
| GadB-F | CCCAAGCTTATGCATCATCACCATCACCACGTACCTATGTTATACGGAAAAG, *Hin*dIII | Cloning of *gadB* expression | *L. lactis* F44 |
| GadB-R | CGGGATCCATGAATATCAGCTTTTTTAGTG, *Bam*HI |
| GadC-F | CCCAAGCTTATGCATCATCACCATCACCACTAAGGAGGTATGATGAATCAAA, *Hin*dIII | Cloning of *gadC* expression | *L. lactis* F44 |
| GadC-R | CGGGATCCCGTATAACATAGGTACATCCTCC, *Bam*HI |
| AtpD (L)-F | CCCAAGCTTATGCATCATCACCATCACCACAACAGGAGGAAAAACATTG, *Hin*dIII | Cloning of *atpD* expression | *L. lactis* F44 |
| AtpD (L)-R | CGGGATCCGAAATCGAATTAATAACCCA, *Bam*HI |
| AtpD(E)-F | CCCAAGCTTATGCATCATCACCATCACCACAAGATGGCTACTGGAAAGAT, *Hin*dIII | Cloning of *atpD* expression | *E. coli* DH5α |
| AtpD(E)-R | CGGGATCCCTCCGATTAAGGCGTTAA, *Bam*HI |
| AtpG(E)-F | CCCAAGCTTATGCATCATCACCATCACCACGAGGAGAAGCTCATGGCC, *Hin*dIII | Cloning of *atpG* expression | *E. coli* DH5α |
| AtpG(E)-R | CGGGATCCCTTTCCAGTAGCCATCTTAAAT, *Bam*HI |
| AtpG(L)-F | CCCAAGCTTATGCATCATCACCATCACCACGGAGGCTAACTAATGGGAGCT, *Hin*dIII | Cloning of *atpG* expression | *L. lactis* F44 |
| AtpG(L)-R | CGGGATCCTTAAAGCGCTGAGGCTCCT, *Bam*HI |
| MurA-F | CCCAAGCTTATGCATCATCACCATCACCAC  ATGATGGATAAAATAATTGTAAAA, *Hin*dIII | Cloning of *murA* expression | *L. lactis* F44 |
| MurA-R | CG GGATCCTTAAACATTAGCCTTCTCCG, *Bam*HI |
| MurG-F | CCCAAGCTTATGCATCATCACCATCACCACAGAAAAACAAACATGCGAAT, *Hin*dIII | Cloning of *murG* expression | *L. lactis* F44 |
| MurG-R | CGGGATCCTTATCTTTTTCACTCATTTATTTAC, *Bam*HI |
| Ldh(Z)-F | CGGGATCCGCATCATCACCATCACCACGGCGTGGCAAGTATTACGGATA, *Bam*HI | Cloning of *ldh* expression | *L. casei* Zhang |
| Ldh (Z)-R | TCCCCCGGGTTGCTTACTTATCAGTGATCGTG, *Sma*I |
| Ldh(L)-F | CGGGATCCGCATCATCACCATCACCACGAGAAAATCATGGCTGATAAACA, *Bam*HI | Cloning of *ldh* expression | *L. lactis* F44 |
| Ldh(L)-R | TCC CCCGGG TAGTTGAAATCTCAACCAACTCTT, *Sma*I |
| Pfk (L)-F | CCCAAGCTTATGCATCATCACCATCACCACATGTATCTCAATTTCGGAGGAC, *Hin*dIII | Cloning of *pfk* expression | *L. lactis* F44 |
| Pfk (L)-R | CGGGATCC TAATTATATATCTCTGCCCTAATTATG, *Bam*HI |
| Pyk(L)-F | CCCAAGCTTATGCATCATCACCATCACCACATCATTATAGGAGAAAAACACAA, *Hin*dIII | Cloning of *pyk* expression | *L. lactis* F44 |
| Pyk(L)-R | CGGGATCCGGCAGGCATTATTGTTTTA, *Bam*HI |
| Pfk(Z)-F | CCCAAGCTTATGCATCATCACCATCACCACTGAGGTGAAATAATGAAACGCA, *Hin*dIII | Cloning of *pfk* expression | *L. casei* Zhang |
| Pfk(Z)-R | CGGGATCCTGTCGTTTGTTTACGAGATTAAAA, *Bam*HI |
| Pyk(Z)-F | CCCAAGCTTATGCATCATCACCATCACCACGGAGCGATTTCACTTATGAAA, *Hin*dIII | Cloning of *pyk* | *L. casei* Zhang |
| Pyk(Z)-R | CGGGATCCTCCTAACCTTTAACAGGTCATTT, *Bam*HI |
| Co-gadB-F | CGGGATCCGCATCATCACCATCACCACGATGTACCTATGTTATACGGAAAAG, *Bam*HI | Cloning of  *gadB* for co-expression | *L. lactis* F44 |
| Co-gadB-R | TTTGATTCATCATACCTCCTTATATTAAGATGAATATCAGCTTTTTTAGTG |
| Co-gadC-F | CACTAAAAAAGCTGATATTCATCTTAATATAAGGAGGTATGATGAATCAAA | Cloning of *gadC* for co-expression | *L. lactis* F44 |
| Co-gadC-R | TCCCCCGGGTTAGTGGTGATGGTGATGATGATGTTTTAAGATATGTTCTTCTTTTTT, *Sma*I |
| Co-HdeA-F | CG GGATCC TATTACGATGAAAAAAGTATTAGGC, *Bam*HI | Cloning of *hdeA* for co-expression | *E. coli* DH5α |
| Co-HdeA-R | GATGAAATATTCATTTTGTAACCCCCTCCTT  ACGCATTCCCGGAATTAC |
| Co-HdeB-F | GTAATTCCGGGAATGCGTAAGGAGGGGGTTACAAAATGAATATTTCATC | Cloning of *hdeB* for co-expression | *E. coli* DH5α |
| Co-HdeB-R | GCATGTTTGTTTTTCTCCAATACTTGCACCTCATTAATTCGG |
| Co-MurG-F | CCGAATTAATGAGGTGCAAGTATTGGAGAAAAACAAACATGC | Cloning of *murG* for co-expression | *L. lactis* F44 |
| Co-MurG-abg-R | TCCCCCGGGGTTATTATCTTTTTCACTCATTTATTTAC, *Sma*I |
| Co-MurG-abgl-R | CCTTTCTTATGTGCATGCAAGTTATTATCTTTTTCACTCATTTATTTAC | Cloning of *MurG* for co-expression | *L. lactis* F44 |
| Co-Ldh-abgl-F | GTAAATAAATGAGTGAAAAAGATAATAACTTGCATGCACATAAGAAAGG | Cloning of *ldh* for co-expression | *L. casei* Zhang |
| Co-Ldh-abgl-R | TCCCCCGGGTTGCTTACTTATCAGTGATCGTG, *Sma*I |
| Co-MurG-mlz-F | CGGGATCCGTATTGGAGAAAAACAAACATGC, *Bam*HI | Cloning of *MurG* for co-expression | *L. lactis* F44 |
| Co-MurG-mlz-R | CCTTTCTTATGTGCATGCAAGTTATTATCTTTTTCACTCATTTATTTAC |
| Co-Ldh- mlz-F | GTAAATAAATGAGTGAAAAAGATAATAACTTGCATGCACATAAGAAAGG | Cloning of *ldh* for co-expression | *L. casei* Zhang |
| Co-Ldh- mlz-R | TCC CCCGGG TTGCTTACTTATCAGTGATCGTG, *Sma*I |
| Co-Ldh-mll-F | CGGGATCCCATGGCTGATAAACAACGTAA, *Bam*HI | Cloning of *ldh* for co-expression | *L. lactis* F44 |
| Co-Ldh-mll-R | GCATGTTTGTTTTTCTCCAATAAGTTGAAATCTCAACCAACTCTT |
| Co-MurG-mll-F | AAGAGTTGGTTGAGATTTCAACTTATTGGAGAAAAACAAACATGC | Cloning of *MurG* for co-expression | *L. lactis* F44 |
| Co-MurG-mll-R | TCCCCCGGGGTTATTATCTTTTTCACTCATTTATTTAC, *Sma*I |
| Co-Pfk-ppl-F | CGGGATCCAATAATGAAACGCATTGGTATT, *Bam*HI | Cloning of *pfk* for co-expression | *L. casei* Zhang |
| Co-Pfk-ppl-R | CATAAGTGAAATCGCTCCTTGATTAAAATGTTAACTCTTCCGCT |
| Co-Pyk-ppl-F | AGCGGAAGAGTTAACATTTTAATCAAGGAGCGATTTCACTTATG | Cloning of *pyk* for co-expression | *L. casei* Zhang |
| Co-Pyk-ppl-R | CCTTTCTTATGTGCATGCAATTCCTAACCTTTAACAGGTCATT |
| Co-Ldh-ppl-F | AATGACCTGTTAAAGGTTAGGAATTGCATGCACATAAGAAAGG | Cloning of *ldh* for co-expression | *L. casei* Zhang |
| Co-Ldh-ppl-R | TCCCCCGGGTTGCTTACTTATCAGTGATCGTG, *Sma*I |
| HdeA-qPCR-F | GTGAAGATTTCCTGGCTGTG | Amplification of *hdeA* for qRT-PCR | *E. coli* DH5α |
| HdeA-qPCR-R | GCAATACCCTGAACATCTAAAA |
| HdeB-qPCR-F | CCGCTAAAGATATGACCTGC | Amplification of *hdeB* for qRT-PCR | *E. coli* DH5α |
| HdeB-qPCR-R | ACGGTATCGCCACCTTTATA |
| MurG-qPCR-F | TTATGTCCCAAGTCCAAATGT | Amplification of *murG* for qRT-PCR | *L. lactis* F44 |
| MurG-qPCR-R | TTGACCCGTTAGTTCTTCGT |
| Ldh(z)-qPCR-F | GTTCTGCCACTTTCCGTTTA | Amplification of *ldh* for qRT-PCR | *L. casei* Zhang |
| Ldh(z)-qPCR-R | GTCAATGGGATTTCCAGGA |
| NisZ-qPCR-F | CATCACCACGCATTACAA | Amplification of *nisZ* for qRT-PCR | *L. lactis* F44 |
| NisZ-qPCR-R | TTTGCTTACGTGAATACTACAA |
| NisB-qPCR-F | TTAGCTTACGGATCTATTCTTG | Amplification of *nisB* for qRT-PCR | *L. lactis* F44 |
| NisB-qPCR-R | CAAATCCACCATATCTTTCTAC |
| NisT-qPCR-F | GCGTCAACTTTCAGGAGG | Amplification of *nisT* for qRT-PCR | *L. lactis* F44 |
| NisT-qPCR-R | GTGCAGCACTTGGTTCATC |
| NisC-qPCR-F | CTTTACATCAAATCGGAGAATC | Amplification of *nisC* for qRT-PCR | *L. lactis* F44 |
| NisC-qPCR-R | CATGTGCTAATCCCATATTCA |
| NisI-qPCR-F | GGGAGAATTGATAAGGATGGT | Amplification of *nisI* for qRT-PCR | *L. lactis* F44 |
| NisI-qPCR-R | ACGGCAAATGCTTCAGTAAGA |
| NisP-qPCR-F | GGAGGGTTTGATAATGAAGAA | Amplification of *nisP* for qRT-PCR | *L. lactis* F44 |
| NisP-qPCR -R | CTGTAATCTGACCTGCGACTT |
| NisR-qPCR-F | GGTATTGGTGGGGATGACTAT | Amplification of *nisR* for qRT-PCR | *L. lactis* F44 |
| NisR-qPCR-R | AACTGCATGTTTATTGCGTTC |
| NisK-qPCR-F | TGGACTATCTTTTGCTCAAGG | Amplification of *nisK* for qRT-PCR | *L. lactis* F44 |
| NisK-qPCR-R | TTAGGATAACTTCTGCCCCAC |
| NisF-qPCR-F | GATGGTATTGCGGAGTTGTTA | Amplification of *nisF* for qRT-PCR | *L. lactis* F44 |
| NisF-qPCR-R | TTATTTCGTGCAACTGATGAC |
| NisE-qPCR-F | TTTCTTATGGGTGGAATACAG | Amplification of  *nisE* for qRT-PCR | *L. lactis* F44 |
| NisE-qPCR-R | GCAAACTCATCAAAAGGAATA |
| NisG-qPCR-F | GGATTTCCTTTTGTTCTTTCC | Amplification of *nisG* for qRT-PCR | *L. lactis* F44 |
| NisG-qPCR-R | ATCATTCCTTGTTGCCCTACT |
| 16S-qPCR-F | GATGATACATAGCCGACCTGA | Amplification of 16S for qRT-PCR | *L. lactis* F44 |
| 16S-qPCR-R | TTCCCTACTGCTGCCTCC | Amplification of 16S for qRT-PCR | *L. lactis* F44 |

F: forward, R: reverse. Co: co-expression, Underline: restriction enzyme site.

**Table S3. Effect of different acid tolerance genes on biomass of strains, pH of the fermentation broth and nisin titer.**

| **Cell growth (OD_600_)** | | | | | | | |
| --- | --- | --- | --- | --- | --- | --- | --- |
| strain | 2h | 4h | 6h | 8h | 10h | 12h | 14h |
| MleS | 0.219±0.022 | 0.571±0.014 | 1.136±0.043 | 1.321±0.099 | 1.330±0.090 | 1.351±0.076 | 1.323±0.041 |
| MleP | 0.224±0.021 | 0.569±0.011 | 1.143±0.017 | 1.309±0.045 | 1.319±0.089 | 1.339±0.019 | 1.309±0.60 |
| CadB | 0.228±0.029 | 0.599±0.018 | 1.153±0.079 | 1.328±0.140 | 1.332±0.079 | 1.351±0.024 | 1.331±0.072 |
| cadA | 0.221±0.021 | 0.621±0.033 | 1.198±0.046 | 1.324±0.097 | 1.339±0.112 | 1.349±0.026 | 1.321±0.026 |
| GadB | 0.216±0.019 | 0.610±0.019 | 1.192±0.061 | 1.319±0.152 | 1.338±0.109 | 1.351±0.089 | 1.335±0.012 |
| GadC | 0.233±0.013 | 0.612±0.035 | 1.178±0.084 | 1.310±0.092 | 1.321±0.053 | 1.359±0.070 | 1.337±0.086 |
| GadBC | 0.258±0.019 | 0.633±0.029 | 1.179±0.053 | 1.214±0.166 | 1.338±0.078 | 1.345±0.068 | 1.334±0.057 |
| MurA | 0.209±0.018 | 0.623±0.022 | 1.201±0.104 | 1.322±0.075 | 1.335±0.15 | 1.356±0.076 | 1.341±0.042 |
| MurG | 0.269±0.024 | 0.667±0.051 | 1.321±0.123 | 1.338±0.118 | 1.354±0.044 | 1.380±0.022 | 1.343±0.096 |
| Cfa | 0.211±0.036 | 0.581±0.032 | 1.196±0.129 | 1.345±0.041 | 1.329±0.107 | 1.328±0.077 | 1.345±0.051 |
| AtpD(L) | 0.224±0.019 | 0.631±0.043 | 1.213±0.060 | 1.299±0.068 | 1.314±0.081 | 1.331±0.113 | 1.321±0.049 |
| AtpD(E) | 0.218±0.007 | 0.600±0.023 | 1.231±0.055 | 1.321±0.094 | 1.341±0.072 | 1.351±0.143 | 1.342±0.086 |
| AtpG(L) | 0.225±0.012 | 0.564±0.045 | 1.137±0.075 | 1.323±0.059 | 1.339±0.075 | 1.296±0.050 | 1.309±0.089 |
| AtpG(E) | 0.221±0.029 | 0.598±0.016 | 1.178±0.075 | 1.332±0.034 | 1.342±0.036 | 1.361±0.060 | 1.350±0.020 |
| HdeA | 0.201±0.013 | 0.579±0.061 | 1.192±0.040 | 1.317±0.079 | 1.321±0.064 | 1.351±0.086 | 1.341±0.141 |
| HdeB | 0.203±0.025 | 0.581±0.055 | 1.165±0.079 | 1.310±0.059 | 1.325±0.136 | 1.348±0.073 | 1.320±0.136 |
| HdeAB | 0.232±0.012 | 0.612±0.039 | 1.211±0.073 | 1.333±0.057 | 1.336±0.053 | 1.354±0.040 | 1.321±0.085 |
| F44 | 0.239±0.030 | 0.666±0.023 | 1.243±0.037 | 1.326±0.157 | 1.306±0.157 | 1.298±0.070 | 1.248±0.101 |
| **pH of fermentation broth** | | | | | | | |
| strain | 2h | 4h | 6h | 8h | 10h | 12h | 14h |
| MleS | 7.06±0.09 | 6.93±0.06 | 6.22±0.08 | 5.21±0.06 | 5.01±0.08 | 4.85±0.07 | 4.76±0.07 |
| MleP | 7.02±0.08 | 6.87±0.12 | 6.21±0.09 | 5.20±0.07 | 5.04±0.05 | 4.83±0.02 | 4.74±0.11 |
| CadB | 7.07±0.11 | 6.90±0.09 | 6.24±0.08 | 5.23±0.07 | 4.99±0.08 | 4.81±0.06 | 4.83±0.09 |
| CadA | 7.02±0.03 | 6.85±0.11 | 6.21±0.12 | 5.25±0.07 | 4.97±0.07 | 4.87±0.09 | 4.82±0.09 |
| GadB | 7.08±0.05 | 6.92±0.08 | 6.24±0.07 | 5.20±0.09 | 4.91±0.08 | 4.85±0.08 | 4.81±0.03 |
| GadC | 7.03±0.13 | 6.90±0.03 | 6.22±0.05 | 5.18±0.09 | 5.00±0.06 | 4.88±0.09 | 4.82±0.08 |
| GadBC | 7.04±0.03 | 6.91±0.06 | 6.20±0.07 | 5.23±0.13 | 4.97±0.08 | 4.88±0.02 | 4.79±0.03 |
| MurA | 7.05±0.17 | 6.83±0.06 | 6.22±0.05 | 5.21±0.08 | 5.01±0.07 | 4.92±0.07 | 4.81±0.09 |
| MurG | 6.94±0.09 | 6.73±0.06 | 5.89±0.15 | 5.00±0.09 | 4.80±0.08 | 4.67±0.10 | 4.61±0.08 |
| Cfa | 7.05±0.04 | 6.93±0.10 | 6.24±0.07 | 5.27±0.14 | 4.99±0.02 | 4.90±0.03 | 4.82±0.08 |
| AtpD(L) | 7.06±0.08 | 6.87±0.08 | 6.22±0.04 | 5.23±0.07 | 5.00±0.06 | 4.88±0.07 | 4.78±0.05 |
| AtpD(E) | 7.07±0.09 | 6.90±0.07 | 6.20±0.07 | 5.21±0.08 | 5.01±0.09 | 4.83±0.09 | 4.76±0.12 |
| AtpG(L) | 7.07±0.07 | 6.92±0.05 | 6.22±0.06 | 5.20±0.07 | 5.02±0.08 | 4.85±0.06 | 4.71±0.11 |
| AtpG(E) | 7.08±0.06 | 6.90±0.07 | 6.21±0.09 | 5.23±0.02 | 5.01±0.06 | 4.81±0.06 | 4.77±0.08 |
| HdeA | 7.04±0.08 | 6.89±0.10 | 6.21±0.09 | 5.26±0.09 | 4.96±0.13 | 4.87±0.05 | 4.80±0.14 |
| HdeB | 7.05±0.04 | 6.90±0.06 | 6.23±0.02 | 5.21±0.12 | 5.01±0.06 | 4.89±0.16 | 4.81±0.13 |
| HdeAB | 7.03±0.04 | 6.91±0.09 | 6.24±0.06 | 5.25±0.07 | 4.99±0.08 | 4.84±0.10 | 4.72±0.09 |
| F44 | 7.01±0.08 | 6.80±0.05 | 6.13±0.07 | 5.17±0.13 | 4.97±0.05 | 4.90±0.08 | 4.82±0.03 |
| **Nisin titer (IU/mL)** | | | | | | | |
| strain | 2h | 4h | 6h | 8h | 10h | 12h | 14h |
| MleS | 221±17 | 467±111 | 2011±412 | 3312±364 | 2500±257 | 1832±230 | 1210±224 |
| MleP | 201±64 | 450±130 | 1523±592 | 3023±507 | 2314±314 | 1763±107 | 1209±147 |
| CadB | 214±86 | 467±278 | 1521±235 | 3111±82 | 2245±415 | 1699±380 | 1234±181 |
| CadA | 229±65 | 443±289 | 1499±457 | 3045±420 | 2300±334 | 1654±336 | 1232±227 |
| GadB | 231±68 | 478±95 | 2210±242 | 3314±423 | 2599±332 | 1910±434 | 1234±210 |
| GadC | 209±32 | 488±25 | 1400±355 | 3156±371 | 2248±230 | 1699±649 | 1219±397 |
| GadBC | 245±65 | 497±131 | 2000±332 | 3657±316 | 2589±413 | 1930±192 | 1299±306 |
| MurA | 221±47 | 478±36 | 1699±692 | 3423±462 | 2431±256 | 1812±602 | 1221±189 |
| MurG | 231±69 | 677±265 | 2411±625 | 4377±394 | 2766±549 | 1787±314 | 1259±263 |
| Cfa | 221±52 | 523±150 | 1558±485 | 3209±461 | 2134±377 | 1822±452 | 1267±201 |
| AtpD(L) | 219±41 | 613±171 | 1812±105 | 3614±474 | 2524±163 | 1910±275 | 1245±280 |
| AtpD(E) | 225±80 | 547±159 | 1656±780 | 3534±326 | 2413±443 | 1842±560 | 1254±444 |
| AtpG(L) | 267±62 | 446±105 | 1897±524 | 4111±518 | 2477±405 | 1699±292 | 1355±148 |
| AtpG(E) | 219±47 | 470±289 | 1656±460 | 3604±566 | 2453±486 | 1899±263 | 1267±256 |
| HdeA | 245±121 | 467±195 | 1890±441 | 3210±675 | 2201±498 | 1800±433 | 1251±259 |
| HdeB | 278±66 | 488±139 | 2066±203 | 3545±409 | 2299±392 | 1867±480 | 1278±296 |
| HdeAB | 300±151 | 510±73 | 2100±246 | 3850±528 | 2459±184 | 2102±226 | 1300±162 |
| F44 | 200±99 | 456±240 | 1489±329 | 2810±253 | 2109±387 | 1614±185 | 1211±238 |

**Supplementary References**

S1. Zhang, Y. F. et al. Genome shuffling of *Lactococcus lactis* subspecies *lactis* YF11 for improving nisin Z production and comparative analysis. *J Dairy Sci* **97**, 2528-2541 (2014).

S2. Ya, T. et al. Immunological evaluation of *Lactobacillus casei* Zhang: a newly isolated strain from koumiss in Inner Mongolia, China. *BMC immunology* **9**, 68 (2008).
